# An inflammatory and quiescent HSC subpopulation expands with age in humans

**DOI:** 10.1186/s13059-026-03936-z

**Published:** 2026-01-16

**Authors:** Ksenia R. Safina, Basit Salik, Dylan Kotliar, Michelle Curtis, Jonathan D. Good, Chen Weng, Shawn David, Soumya Raychaudhuri, Antonia Kreso, Jennifer J. Trowbridge, Vijay G. Sankaran, Peter van Galen

**Affiliations:** 1 Division of Hematology, Brigham and Women’s Hospital, Boston, MA 02115 USA; 2 Department of Medicine, Harvard Medical School, Boston, MA 02115 USA; 3 Broad Institute of MIT and Harvard, Cambridge, MA 02142 USA; 4 Ludwig Center at Harvard, Boston, MA 02115 USA; 5 Division of Rheumatology, Inflammation, and Immunity, Department of Medicine, Brigham and Women’s Hospital and Harvard Medical School, Boston, MA 02115 USA; 6 Center for Data Sciences, Brigham and Women’s Hospital and Harvard Medical School, Boston, MA 02115 USA; 7 Division of Hematology/Oncology, Boston Children’s Hospital, Harvard Medical School, Boston, MA 02115 USA; 8 Whitehead Institute for Biomedical Research, Cambridge, MA 02142 USA; 9 Department of Pediatric Oncology, Dana-Farber Cancer Institute, Harvard Medical School, Boston, MA 02115 USA; 10 Howard Hughes Medical Institute, Boston, MA 02115 USA; 11 The Jackson Laboratory, Bar Harbor, ME 04609 USA; 12 The University of Maine, Orono, ME USA; 13 Department of Biomedical Informatics, Harvard Medical School, Boston, MA 02115 USA; 14 Department of Surgery, Massachusetts General Hospital, Boston, MA 02115 USA; 15 Cardiovascular Research Center, Massachusetts General Hospital, Boston, MA 02115 USA; 16 Harvard Stem Cell Institute, Cambridge, MA 02138 USA

## Abstract

Aging of the blood system impacts systemic health and can be traced to hematopoietic stem cells (HSCs). Despite multiple reports on human HSC aging, a unified map detailing their molecular age-related changes is lacking. We developed a consensus map of gene expression in HSCs by integrating seven single-cell datasets. This map reveals previously unappreciated heterogeneity within the HSC population. It also links inflammatory pathway activation (TNF/NFκB, AP-1) and quiescence within a single gene expression program. This program dominates an inflammatory HSC subpopulation that increases with age, highlighting a potential target for further experimental studies and anti-aging interventions.

**Supplementary Information:**

The online version contains supplementary material available at 10.1186/s13059-026-03936-z.

## Background

The aging blood system has a decreasing capacity to mount effective immune responses, transport oxygen, and produce lymphocytes [1–3]. Many of these changes can be traced to HSCs, which exhibit age-associated clonal expansion and myeloid bias [4–7]. Human studies across age groups have revealed multiple alterations including epigenetic reprogramming, decreased polarity, and skewed differentiation [8–11]. However, there is little consensus on the molecular programs consistently altered with age, in part due to technical variation across datasets [12]. Moreover, while the human HSC compartment is often analyzed as a whole, heterogeneity can be observed using single-cell sequencing [13, 14]. The molecular basis of this heterogeneity, and how it evolves with age, remains poorly defined. To establish a robust reference for HSC aging and define the extent to which HSC heterogeneity changes with age, we undertook an effort to integrate human HSC aging studies. Our analysis reveals a consensus program of inflammation and quiescence in a subpopulation of HSCs that becomes increasingly dominant with age.

## Results and discussion

**Fig. 1:**
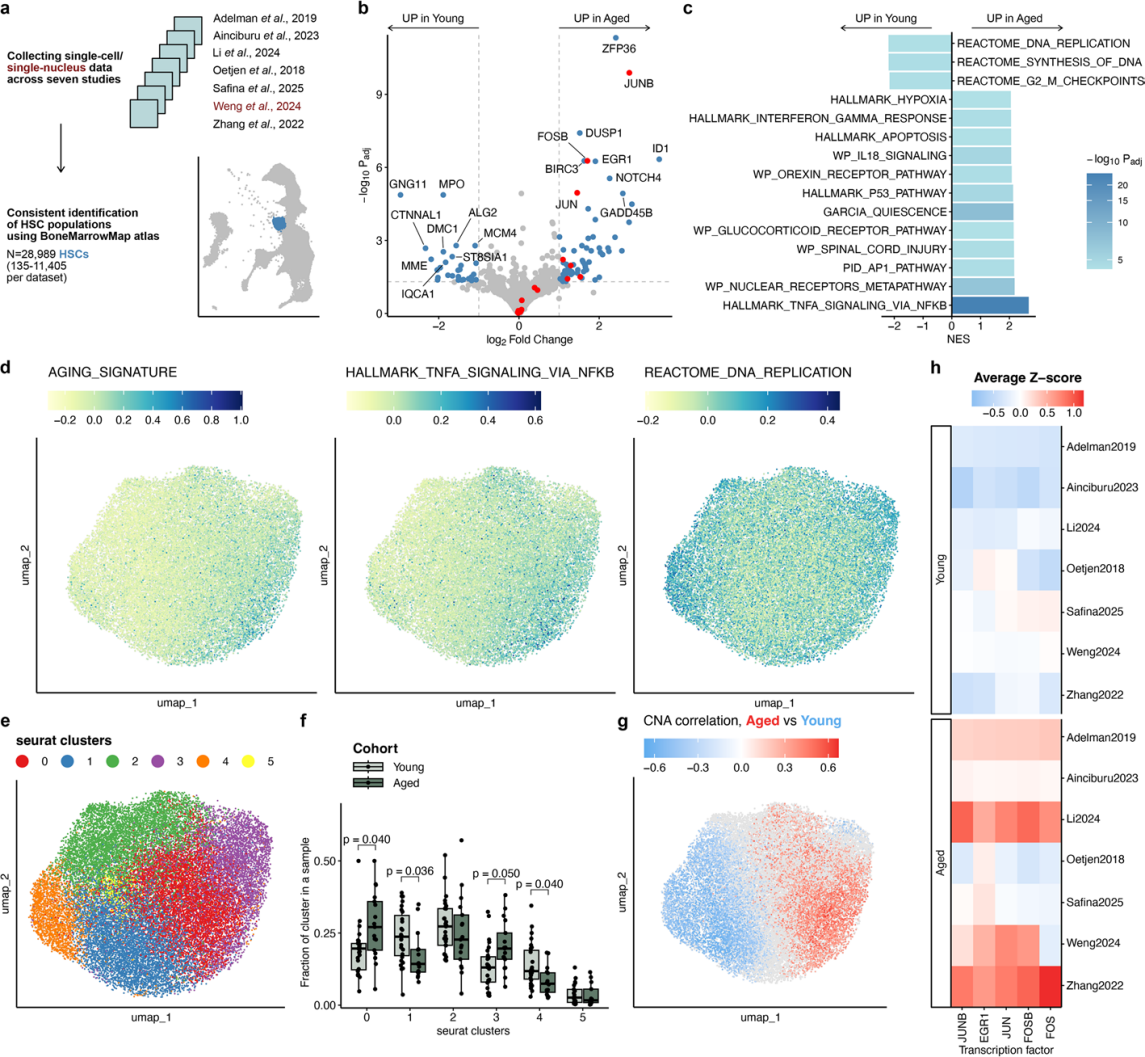
Data from multiple single-cell datasets show that transcriptional heterogeneity of human HSCs changes with age. **a** Schematic shows dataset collection and HSC annotation. **b** Volcano plot shows the differentially expressed genes in Young vs Aged samples across five datasets. AP-1 members are shown in red, genes with absolute logFC > 1 and p.adjusted < 0.05 are shown in blue; top-10 most significant hits per cohort and significant AP-1 members are labeled. **c** Bar plot shows top-15 most significant gene sets in GSEA analysis; NES, normalized enrichment score. **d** Uniform Manifold Approximation and Projections (UMAPs) show all integrated HSCs, colored by score for aging signature, Hallmark TNF-α/NF-κB signaling gene set, and Reactome DNA replication gene set. **e** UMAP shows HSC clusters identified in Seurat. **f** Box plot shows comparison of cluster abundances between the Young (*n* = 27) and Aged (*n* = 17) cohorts. Symbols indicate cluster fractions per sample; *p*-values of two-sided Wilcoxon test with Bonferroni correction are shown. **g** UMAP is colored by neighborhood coefficient of correlation to Aged vs. Young cohorts; neighborhoods that didn’t pass FDR < 10% are colored in gray. **h** Heatmap shows activity of the top-5 regulons associated with the Aging cohort, identified by SCENIC; averaged Z-scores of AUC values are shown

To robustly characterize age-associated changes in human HSCs, we combined single-cell/single-nucleus data across six studies and one unpublished dataset from 98 individuals, and annotated 28,989 HSCs using the BoneMarrowMap atlas (Fig. 1a, Additional file 1: Table S1). An orthogonal approach of identifying and annotating clusters on the integrated object yielded 44,187 HSCs and included 87% of HSCs identified by BoneMarrowMap (Additional file 2: Fig. S1), suggesting that the BoneMarrowMap algorithm selects an accurate and stringent population of HSCs. All downstream analysis was performed on BoneMarrowMap-annotated HSCs.

First, we inferred differential gene expression (DE) between young and aged samples with at least 20 HSCs (young: 19–37 y.o., *n* = 25; aged: 60–87 y.o., *n* = 15). 68 and 29 genes were up- and down-regulated in aged samples, respectively (Fig. 1b, Additional file 1: Table S2, a). Notably, three members of the AP-1 complex, *JUN, JUNB,* and *FOSB*, implicated in stress-activated MAPK signaling and quiescence, were consistently upregulated across studies (Additional file 2: Fig. S2) [15–17]. Our DE results broadly agree with prior bulk RNA-seq-based reports and stress the importance of meta-analyses integrating data across multiple independent studies (Additional file 2: Figs. S3, S4).

Gene set enrichment analysis (GSEA) revealed two major biological phenomena distinguishing expression profiles of young and aged samples (Fig. 1c, Additional file 1: Table S2, b). First, several inflammatory-response pathways, including TNF via NFκB, IFN-g response, and AP-1 signaling, were upregulated in aged samples. Second, aged samples showed higher quiescence, in contrast to young samples showing more active proliferation. These findings are in agreement with previous reports showing increased circulating TNF and delayed cell cycle entry of aged HSCs [9, 18, 19].

The activity of these pathways was not uniform across the population of HSCs (Fig. 1d, Additional file 2: Fig. S5, a). The aging signature (defined as 68 genes up-regulated in aged samples), together with TNF signaling, was more active in clusters 0 and 3 (Fig. 1e, Additional file 2: Fig. S5, b). These clusters were more abundant in Aged samples (Fig. 1f). DNA replication, E2F, and cell cycle signatures were most active in cluster 4, one of two clusters that were more abundant in Young samples (Additional file 2: Fig. S5, b, c). Differential abundance of HSC populations between the Young and Aged cohorts was supported by cluster-independent co-varying neighborhood analysis (CNA, Fig. 1g, Additional file 2: Fig. S5, d). To explore this heterogeneity further, we next inferred the activity of transcription factors (TFs) using SCENIC. Despite substantial variability between datasets and individuals, combined analysis highlighted several AP-1 members as the top differentially active TFs in the aged cohort (Fig. 1h, Additional file 2: Fig. S6), in agreement with DE results. Altogether, these observations suggest that at least part of the observed transcriptional heterogeneity in HSCs can be attributed to age, prompting us to investigate its potential sources.

**Fig. 2:**
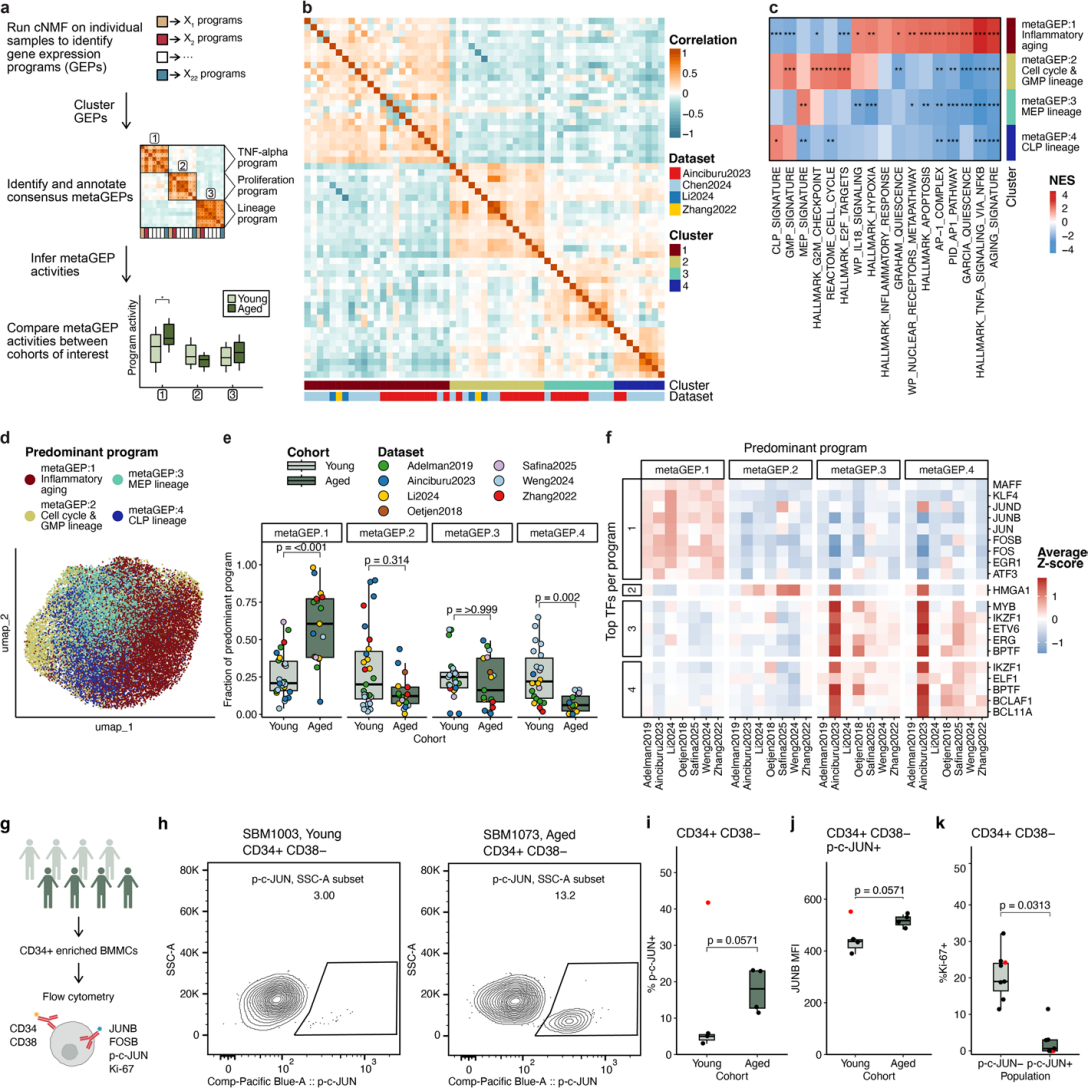
cNMF analysis recovers four gene expression meta-programs (metaGEPs) in human HSCs, including an age-dependent program. **a** Schematic shows cNMF analysis steps. **b** Heatmap shows Pearson correlation between Z-score vectors of individual programs comprising the four identified metaGEPs. **c** Heatmap shows relevant pathways enriched in metaGEPs as inferred by GSEA; BH-corrected *p*-values: *, *p* < 0.05, **, *p* < 0.01, ***, *p* < 0.001. **d** UMAP shows all integrated HSCs, colored by the predominantly active metaGEP. **e** Box plot shows comparison of predominant program abundances between the Young (*n* = 27) and Aged (*n* = 17) cohorts. Symbols indicate cluster fractions per sample; *p*-values of two-sided Wilcoxon test with Bonferroni correction are shown. **f** Heatmap shows activity of metaGEP-specific regulons, identified by SCENIC; averaged Z-scores of AUC values are shown. GEP: gene expression program, GMP: granulocyte–macrophage progenitor, MEP: megakaryocyte-erythrocyte progenitor, CLP: common lymphoid progenitor. **g** Schematic shows steps to measure FOS, p-c-JUN (S63), and JUNB levels in human HSPCs. **h** Representative flow cytometry plots show p-c-JUN staining in CD34+ CD38– HSPCs from a young (left) and aged (right) donor. Full gating is shown in Additional file 2: Fig. S10. **i** Boxplot shows the percent p-c-JUN+ cells within the CD34+ CD38– HSPC compartment of four young and four aged donors. **j** Boxplot shows the mean fluorescence intensity (MFI) of JUNB in CD34+ CD38–p-c-JUN+ HSPCs in young vs. aged samples. **k** Boxplot shows the percentage of Ki-67-positive cells in p-c-JUN+ and p-c-JUN– CD34+ CD38– populations. **i**-**k** the red sample (SBM1048) was excluded from statistical testing due to potential inflammatory signaling related to May-Thurner syndrome (see Methods). **i**, **j** *P*-values were calculated using the Wilcoxon test. **k** *P*-value was computed using the paired Wilcoxon test

To capture heterogeneity across cells that may be missed by conventional DE analysis and clustering, we used consensus non-negative matrix factorization (cNMF). This cluster-free and unbiased approach decomposes each cell’s gene expression profile into a set of underlying gene expression programs (GEPs) active across cells in the experiment (Fig. 2a) [20]. We ran cNMF on 22 samples with at least 100 HSCs (*n* = 14 young, *n* = 8 aged) to identify GEPs reproducible across datasets. We then clustered GEPs from individual samples together, which identified four representative clusters carrying at least four programs coming from at least two datasets, yielding four meta-programs (metaGEPs) (Fig. 2b, Additional file 2: Fig. S7, Additional file 1: Table S3). To functionally annotate metaGEPs 1–4, we assessed the enrichment of relevant gene sets in each of the meta-programs (Fig. 2c). metaGEP1 was enriched for the aging signature, TNF signaling, and quiescence, indicating that the activity of these processes is linked within a single program; we termed metaGEP1 the inflammatory aging program. The three remaining programs were mainly associated with lineage commitment and were annotated accordingly. metaGEP2 was enriched for a granulocyte–macrophage progenitor (GMP) signature and for cell-cycle-related pathways. metaGEPs 3–4 showed enrichment for megakaryocyte-erythrocyte progenitor (MEP) and common lymphoid progenitor (CLP) signatures, respectively (Fig. 2c).

Similar to our prior DE-based observations (Fig. 1d, Additional file 2: Fig. S5, a, b), the activity of metaGEPs was not uniform across the population of HSCs and suggested that HSCs are composed of subpopulations of cells governed by different programs (Additional file 2: Fig. S8). To define these HSC subpopulations, we identified the predominant program in each cell of the original integrated object (Fig. 2d). To investigate whether the activity of metaGEPs 1–4 changes with age, we compared the fractions of cells predominated by each of the four programs in every sample between the young and aged cohorts with at least 20 HSCs (*n* = 27 young, *n* = 17 aged, Fig. 2e). Inflammatory aging metaGEP1 was more abundant in aged samples, while CLP-associated metaGEP4 showed higher activity in the young cohort. The latter agrees with lower lymphoid output with age [1, 9, 21]. Overlaying metaGEP activities with TF activities inferred by SCENIC revealed nine TFs whose activity was highly and consistently correlated with metaGEP1 usages across the datasets, including seven AP-1 complex members and two TFs implicated in maintaining quiescence, KLF4 and EGR1 [22, 23] (Fig. 2f, Additional file 2: Fig. S9). These findings indicate metaGEP1 underlies age-associated differences in human HSCs and reveal molecular features of an inflammatory and quiescent HSC population that expands with age.

To assess AP-1 protein levels in young and aged CD34+ CD38– HSPCs, we performed intracellular flow cytometry on FOSB, JUNB, and phospho-c-JUN at serine 63 (p-c-JUN), an accepted activated form of c-JUN [24] (Fig. 2g, Additional file 2: Figs. S10, S11). We did not observe significant differences in FOSB and JUNB protein levels between young and aged HSCs (Additional file 2: Fig. S11). This result is distinct from what we observed at the transcript level with reproducibly increased FOSB and JUNB in aged HSCs. While this may reflect differences in cell processing [25, 26], we propose that the transcript differences remain biologically meaningful, as it implies that aged HSCs are more prone to stress and respond differently than young cells. Additionally, we found a trend towards an increased p-c-JUN+ population in the aged HSPC compartment, suggesting a possible increase in AP-1 activity (Fig. 2h, i, Additional file 2: Fig. S12). These p-c-JUN+ HSPCs tended to have increased JUNB levels in aged compared to young samples and were predominantly quiescent (Fig. 2j, k, Additional file 2: Fig. S13). Further experimentation will be required to dissect the functional consequences of AP-1 signaling in young and aged HSCs.

In this study, we aggregated data from seven single-cell and single-nucleus studies and consistently annotated 28,989 HSCs across individuals, including 27 young and 17 aged samples with at least 20 HSCs, and identified four meta-programs that drive transcriptional heterogeneity through variable activity across HSCs. The largest meta-program we recovered, metaGEP1, was enriched for TNF signaling and quiescence, and marked a subpopulation of HSCs that expanded with age. These findings indicate that HSC aging is not uniform but is instead shaped by shifting program activities.

Inflammation has been linked to HSC activation and exhaustion [27–29]. In contrast, our data link inflammatory signaling and quiescence within a single program associated with aging, more reminiscent of chronic inflammation which can induce quiescence [30, 31]. With the emerging concept that acute inflammatory stimuli induce trained immunity in HSCs [13, 32, 33], an outstanding question is to what extent metaGEP1-dominated HSCs generate pro-inflammatory immune cells that contribute to aging.

Using a mouse HSC aging signature by Svendsen et al*.* [34], overrepresentation analysis indicated overlapping pathways with our human HSC aging GSEA (Fig. 1c). These included TNF signaling, TP53 signaling, hypoxia, and apoptosis (Additional file 2: Fig. S14). Retinoic acid, previously implicated in mouse HSC dormancy [35], was also overrepresented. This potentially indicates a common axis of HSC aging in humans and mice.

In addition to inflammatory signaling and quiescence, aged samples are enriched for TP53 signaling (Fig. 1c), raising a question of whether permanent cell cycle arrest, i.e. a senescent cell state, is driving the signal of quiescence. Analysis of senescence-related pathways (see Additional file 3) indicates that the enrichment of the quiescence signal in our data can not be exclusively attributed to senescence, and quiescence is a more robust explanation of the patterns we observe, even though we cannot fully rule out the age-associated quiescence/senescence switch described in other tissues [36].

Members of the AP-1 complex were among the top-enriched transcription factors in both DE and cNMF analysis. While the activity of immediate early response (IER) genes, including members of AP-1, can be prone to technical variation due to sample processing [25], our finding that AP-1 genes are consistently upregulated across several datasets implies biological relevance. A recent study reported that AP-1-linked chromatin opening with age hijacked the activity of cell identity transcription factors across multiple mouse tissues, reshaping the transcriptome with age [37]—a mechanism that may also be triggered in human HSCs by elevated activity of AP-1 factors. Along with other genes highlighted in our analysis, including anti-inflammatory ZFP36 and NFKBIA [38, 39], perturbation of these factors may modulate HSC aging.

The cluster-independent framework we used to discover the biology of aging HSCs can be easily adapted to larger datasets. Indeed, there were only 22 samples with > 100 HSCs from which to discover GEPs, and larger samples tended to resolve more programs successfully (Additional file 2: Fig. S15). Analyzing more samples with larger cell counts will improve the numerical stability of NMF solutions and may allow for more granular resolution of HSC programs. Finally, since inflammatory pathways in HSCs are affected by clonal hematopoiesis [14], future work should assess how genetic changes interact with the programs identified here.

## Conclusions

Our study provides a consensus, single-cell resolution map of human HSCs by integrating seven prior datasets. Among the most robust changes in aging HSCs are upregulation of pathways associated with inflammatory signaling (TNF, IFN-g), stress-activated MAPK signaling (AP-1), and quiescence. We use a cluster-free approach to uncover activity programs that vary in intensity across individual HSCs. The strongest program shaping HSC heterogeneity is associated with inflammation and quiescence, linking these properties together in individual cells. An inflammatory HSC subset dominated by this program expands with age. These findings open new directions for testing whether modulating inflammatory HSCs can rejuvenate blood production and help maintain healthy hematopoiesis with age.

## Methods

### Single-cell sequencing

We generated one of the seven single-cell RNA-seq datasets ourselves (others were publicly available). Bone marrow cells from nine donors were collected from the iliac crest of patients undergoing cardiac surgery under an excess sample banking and sequencing protocol approved by the Mass General Brigham Institutional Review Board (IRB protocol 2020P004103) and conducted in accordance with the Declaration of Helsinki. All study procedures were covered by the protocol, and participants provided written informed consent prior to inclusion in the study, including consent for publication of de-identified data. Donors were confirmed negative for common CHIP mutations using targeted sequencing. Mononuclear cells were isolated using Ficoll or lymphoprep and cryopreserved in liquid nitrogen storage. Cells were thawed using standard procedures, and viable (DAPI negative) cells were sorted on a Sony SH800 flow cytometer. Next, 10,000–15,000 cells were loaded onto a 10 × Genomics chip. Further processing was done using the recommended procedures for the 10 × Genomics 3’ v3.0, v3.1, or v4 chemistry. Libraries were sequenced on the NovaSeq SP 100 cycle with the following parameters (Read 1: 28 + Read 2: 75 + Index 1 (i7): 10 + Index 2 (i7): 10). Count matrices were generated using CellRanger v.7.1.0 with default settings and GRCh38 as the reference genome. Sequencing data were deposited to NCBI Gene Expression Omnibus under accession number GSE302126 [40].

### Dataset preparation and annotation

We compiled six publicly available human single-cell datasets and added nine samples from our lab. The complete dataset contained bone marrow cells from 98 individuals (9 prenatal, 2 cord blood, 4 infant, 7 child, 31 young, 15 middle-aged, and 30 aged) [4, 11, 41–44]. Gene expression matrices were available for each of the datasets, except for the Adelman and some samples in the Weng datasets. For the Adelman dataset, we mapped sequencing data onto hg38 using STAR [45] with default settings and quantified gene counts using featureCounts from Rsubread package [46] to produce a gene expression matrix. For the Weng dataset, we prepared the input object as described in Github repository petervangalen/ReDeeM_2024. For each of the seven datasets, we loaded the count matrix into Seurat [47], keeping genes captured in at least five cells and cells with at least 100 genes captured. We then filtered the initial matrix based on nCount, nGene, mitochondrial genes content, and doublet scores (as determined by scrubletR [48]) for each sample in the dataset, removing differentiated cell populations where applicable. The accession codes and dataset-specific filtering parameters are available in Additional file 1: Table S1. We then subsetted each of the Seurat objects for genes present in all of the datasets (*n* = 11,612). To obtain concordant cell type annotations among the datasets, we mapped each of the datasets onto the BoneMarrowMap atlas and transferred cell type labels [49]. For subsequent analyses, we only retained cells annotated as HSCs. 30,070 cells were annotated as HSCs; of those, 1,081 cells were mapped outside the reference HSC cluster and were excluded from the analysis, leaving us with 28,989 HSCs.

To cross-validate BoneMarrowMap-based HSC assignment, we integrated seven HSPC datasets with scVI [50] (v1.3.0, n_layers = 4, n_latent = 30, max_epochs = 60), specifying sample name as a batch key and dataset name and single-cell vs. single-nucleus data type as categorical covariates (batch_key = 'Sample', categorical_covariate_keys = ['Dataset', 'data_type']), uploaded the integrated object to Seurat, computed neighborhood graph on scVI latent variables, identified Louvain clusters (resolution = 1.5) and computed UMAP. We scored each cell by three published HSC signatures [51–53], computed average signature scores per cluster (aggregating across cells and three public signatures), and selected four clusters with the highest average HSC score as HSCs. This yielded 44,187 cells and included 87% of HSCs identified by BoneMarrowMap, implying the two annotation approaches are concordant, with BoneMarrowMap being more specific.

### Differential expression analysis

To identify gene expression changes associated with age, we pseudobulked HSC samples per sample and ran DESeq2 [54] using dataset name and sample sex as covariates. Race and ethnicity were not reported in publicly available datasets and therefore not included as covariates. To ensure the robustness of the analysis, we only used samples with at least 20 cells and datasets with at least two such samples in both Young and Aged cohorts (*n* = 25 young and *n* = 15 aged samples across five datasets). We obtained results for the ‘Cohort_Aged_vs_Young’ coefficient and shrunken log fold changes using apeglm [55]. To define the aging signature, we used genes with logFC > 1 and p.adj < 0.05, yielding 68 genes.

### Gene set enrichment analysis

We used the fgsea package [56] to conduct gene set enrichment analysis (GSEA) and characterize ranked gene lists produced in this study. We used two collections of signatures from MSigDB [57–59], HALLMARK and C2:CP. We obtained a quiescence signature from García-Prat et al*.* by selecting genes with logFC < −2 and FDR < 0.00001 from the DE results of Table S1 therein [60]. To define MEP, GMP, and CLP lineage signatures, we identified markers of MEP, Early GMP, and CLP populations of the BoneMarrowMap-annotated object, respectively (using Seurat’s FindMarkers function, providing HSC, MPP-MkEry and MPP-MyLy as ident.2). For each lineage, we selected top-50 genes with p.adj < 1e-10 and logFC > 1, and excluded genes overlapping between the three 50-gene sets. To annotate cNMF metaGEPs, we also included the aging signature (68 genes) identified in the DE analysis of this study. To characterize the recovery of the aging signature compared to a random signature, we generated 20 random signatures with an expression pattern similar to the aging signature and included them in GSEA (as detailed below).

### Comparison with HSC aging signatures from prior bulk studies

To assess the agreement of the aging signature generated in this study with the signatures reported previously, we collected age-associated signatures generated on bulk transcriptomic data in four prior studies: Adelman2019 [11] (we used single-cell RNA-seq data from this study in our analysis, but the authors also generated and analyzed bulk RNA-seq data), Pang2011 [8], Nilsson2016 [61], and Hennrich2018 [62] (this study analyzed CD34+ HSPCs rather than HSCs). For each study, we collected genes significantly up- and down-regulated with age into separate lists (e.g. ADELMAN2019_UP and ADELMAN2019_DOWN) and investigated the agreement of these lists with our DE results on volcano plots (Additional file 2: Fig. S3). We also performed gene set enrichment analysis, which showed that age-associated signatures broadly agree with our DE results (Additional file 2: Fig. S4).

### Variable genes identification

To identify variable genes shared across the seven datasets, we first excluded ribosomal genes (*n* = 94 genes starting with RPS or RPL), then identified top 3,000 variable features within each dataset using FindVariableFeatures() in Seurat, and finally, selected the genes identified as variable in at least 4 datasets out of 7 (*n* = 1,554). We then subsetted the expression count matrix for these genes and used it as an input for scVI and cNMF.

### Dataset integration

We integrated datasets using scVI (v1.3.0, n_layers = 3, n_latent = 30), specifying sample name as a batch key and dataset name as a categorical covariate (batch_key = 'Sample', categorical_covariate_keys = ['Dataset']), uploaded the integrated object to Seurat, computed neighborhood graph on scVI latent variables, identified Louvain clusters (resolution = 0.5) and computed UMAP. Louvain clusters and UMAP coordinates were transferred to the original object with the entire gene set (11,612 genes), to score cells for gene sets of interest using AddModuleScore() (Fig. 1d, Additional file 2: Fig. S5, a).

### Consensus non-negative matrix factorization for inference of gene expression programs

We used consensus non-negative matrix factorization (cNMF) [20] to identify gene expression programs (GEPs) across datasets. To mitigate the effect of sample-driven batch effects, we identified GEPs at the level of individual samples and clustered the discovered programs to define meta-programs (metaGEPs) shared across multiple samples and datasets, similar to the approach taken by Gavish et al. [63]. We used young or aged samples with at least 100 cells, resulting in 8 Ainciburu, 6 Weng, 7 Li, and 1 Zhang sample (total *n* = 22). As cNMF is sensitive to cells with low gene counts and low number of genes, we additionally preprocessed each of the samples as follows. We preserved cells with at least 50 captured genes and the total number of transcripts within the sample-specific limits (see below), and genes expressed in at least 10 cells. Sample-specific total count limits were defined as median ± 2.5 mean absolute deviation for each sample except sample BM3 in Zhang dataset. Data from Zhang et al*.* has an order of magnitude higher expression counts than other datasets with several cells having much higher counts than the median; these outlier cells drive individual programs in cNMF results, dominating the observed variation in expression data. To address this, we used a fixed interval for total counts of [100–10,000] for Zhang sample BM3.

After preprocessing, we ran cNMF on each sample, with 500 iterations of factorization and a *k* (the number of inferred GEPs) ranging from 3 to 10. For each sample, a single final value of *k* was selected based on the stability vs. error plot and visual inspection of 500 clustered factorization results. We assessed distance thresholds of 0.05, 0.1 and 0.15; distance threshold of 0.1 produced the most visually stable clusters and was used for all the samples. Among the smaller samples, individual cells sometimes drive some of the identified programs, similar to sample BM3 in Zhang dataset. To address this, for each program, we computed the ratio between 100 and 75% quantiles of usage values, and excluded programs with a ratio of more than 10 (which reflects that the maximum usage value is much larger than most usage values). After this filtering, 97 GEPs remained.

### Identification and analysis of metaGEPs

We clustered Z-score spectra of 97 GEPs using the iterative clustering algorithm implemented in starCAT [64] with a modification allowing for more than one program per sample in a cluster (corr_thresh = 0.1, pct_thresh = 0.1). We defined meta-programs (metaGEPs) as clusters carrying at least four programs coming from at least two datasets. Z-score spectra reported by cNMF for each inferred program were averaged within each metaGEP and annotated with GSEA. The variance-normalized TPM spectra within each metaGEP were averaged to produce the meta-program expression matrix (4 metaGEP × 11,612 genes) which we used to infer metaGEP usages in each of the datasets with starCAT [64]. To compare usages between the young and aged cohorts, we defined a single predominant metaGEP per cell, computed the fractions of each predominant metaGEP in every sample with at least 20 cells (*n* = 27 young and *n* = 17 aged samples), and compared the fractions between the two age cohorts.

The aging signature was the most consistently recovered gene set, as it showed the most frequent significant enrichment by GSEA on GEPs. To test whether this high recovery rate is specific to the aging signature rather than its gene expression pattern, we constructed 20 random gene sets and assessed their recovery by GSEA. To construct a random gene set, we computed average normalized expression for 11,612 genes, split the genes into 50 equidistant expression bins, and randomly sampled 68 genes from the bins containing aging signature genes, to mimic the expression pattern of the aging signature. To assess the recoverability of gene sets by GSEA, we revisited our cNMF results, selected all visually stable cNMF runs (*k* values with distance thresholds producing the most stable clusters), and annotated them with GSEA using 20 random gene sets, the aging, TNF signaling, quiescence signature, and the three lineage signatures, applying a Bonferroni correction (which is more stringent than the default Benjamini–Hochberg procedure). For each cNMF run within each sample, we assessed whether it contains a program significantly positively enriched for any of the tested gene sets, and computed the fraction of cNMF runs within each sample that recovered each of the gene sets (Additional file 2: Fig. S15, a). The aging signature was indeed the most recoverable; lineage signatures were less recoverable, but invariably still more recoverable than the random gene sets. We also assessed the recovery rate of a combination of gene sets (Aging + TNF + Quiescence, and Aging + TNF, Additional file 2: Fig. S15, b), which also showed higher recovery than random gene sets. Samples with more HSCs had higher recovery rates than samples with fewer HSCs. This demonstrates that the genesets we used to annotate metaGEPs are robust compared to random gene sets, supporting biological significance, and that using additional large (CD34+ enriched) samples may help resolve and annotate gene expression programs better.

### SCENIC analysis

We used pySCENIC to infer the activity of transcription factors (TFs) [65]. We followed the standard pipeline with default parameters, except using ‘–auc_threshold 0.01’ for the ‘ctx’ command. We inferred TF activities (represented as area under the curve values, AUC) within each dataset separately and then retained 108 TFs identified in all seven datasets. To identify age-specific TFs, we first subsetted young and aged samples, converted age cohort to a binary variable (with 1 being Aged), and computed point-biserial correlation between the binary age variable and TF activities within each dataset. We then assigned ranks to TFs within each dataset, averaged both ranks and correlation values, and selected TFs with mean rank < = 10 and mean correlation > = 0.15 as TFs associated with age, yielding five TFs (Fig. 1h). We repeated the same procedure to infer TFs associated with each of the metaGEPs, retaining samples from all age cohorts and computing Pearson correlation between TF activities and metaGEP usages, instead of point-biserial correlation (Fig. 2f, Additional file 2: Fig. S9). For visualization purposes, AUC values were converted into Z-scores. We limited the Z-score values to the 0.1 and 99.9 percentiles when visualizing TF activities in individual cells (Additional file 2: Fig. S6) to avoid distorting the color scale with outliers.

### Co-varying neighborhood analysis

To test whether the age cohort is associated with certain neighborhoods of cells, we conducted association testing using co-varying neighborhood analysis (CNA) [66]. We first subsetted the integrated object for young and aged samples, encoded age as a binary variable (with 1 being Aged), computed a neighborhood graph using scVI latent variables, and ran CNA, correcting for the dataset name as a batch variable. To visualize significant associations in the UMAP, we colored neighborhoods with FDRs > 0.1 grey. A subpopulation of cells associated with young neighborhoods was visually disconnected from the rest of cells with young neighborhoods (Fig. 1g). See revision/5_explore_umap_cna_discrepancy.ipynb on Github where we explore this observation and find that it likely reflects limitations of the projection algorithm rather than a separate cell state.

### AP-1 levels assessment by intracellular flow cytometry

Bone marrow mononuclear cells for flow cytometry analysis were collected as described under “Single-cell sequencing”. Cryopreserved cells were thawed and assessed for total cell counts and initial viability using a Cellometer Ascend Automated Cell Counter (Revvity, CMT-ASD). Bone marrow cells were then enriched for CD34+ cells using the EasySep Human CD34 Positive Selection Kit II (STEMCELL Technologies, 17856) and an EasyEights EasySep Magnet (STEMCELL Technologies, 18103) using a single magnetic selection step. Following enrichment, cells were kept on ice for sequential staining. Between steps, cells were centrifuged at 300 g for 5 min, washed with PBS containing 2% FBS (FACS buffer) and stored at 4 °C unless otherwise noted. Cells were first stained for viability using Zombie Aqua Fixable Viability Dye (BioLegend, 423101; 1:1000 dilution in PBS) for 15 min at room temperature. Surface staining was then performed for 30 min at 4 °C using APC-eFluor 780-conjugated anti-CD34 (clone 4H11; Invitrogen, 47–0349-42), and PE/Cy7-conjugated anti-CD38 (clone HIT2; BioLegend, 303515). Cells were then fixed and permeabilized using the eBioscience™ Foxp3/Transcription Factor Staining Buffer Set (Invitrogen, 00–5523-00) according to the manufacturer’s instructions. This involved incubating cells in 1X fixation buffer for 30 min at room temperature followed by two washes in 1X permeabilization buffer. Cells were then stained in 1X Permeabilization Buffer with intracellular antibodies for 30 min at room temperature. The intracellular panel included Alexa Fluor 647-conjugated anti-Ki-67 (clone B56 BD Pharmingen, 558615), PE-conjugated anti-FosB (clone W19011B; BioLegend, 600857), FITC-conjugated anti-JunB/AP-1 (Clone PCRP-JUNB-3G2; Novus Biologicals, NBP3-08391F), and mFluor Violet 450 SE-conjugated anti-c-jun [p Ser63] (clone 1018Y; Novus Biologicals, FAB8930MFV450). Compensation was performed using UltraComp eBeads Plus Compensation Beads (Invitrogen, 01–3333-42) alongside unstained and fluorescence-minus-one (FMO) controls. Data were acquired on a BD FACSymphony A3 Cell Analyzer and analyzed with FlowJo v10.

One of the young patients, SBM1048, displayed abnormal levels of phospho c-JUN. We found that this patient had May-Thurner syndrome and leg swelling. The vein blockage is likely to have led to venous hypertension, which results in neutrophil activation and inflammatory signaling [67]. Indeed, inflammatory cytokines IL-1a, IL-6, and MCP-1/CCL2 increased approximately 6-fold in a mouse model of May-Thurner syndrome [68]. Since this may have affected HSPCs, similar to a recent report of HSPC activation following systemic stress [69], we excluded SBM1048 from statistical testing, but included it in boxplots for transparency (red symbols, Fig. 2i-k and Additional file 2: Figs. S10-S13).

### Senescence analysis

While our data indicate robust age-associated activation of genes related to quiescence, the enrichment of TP53 pathway in aged samples points at possible induction of senescence and permanent cell cycle arrest in aged cells. To investigate possible contribution of senescence to age-associated findings in our data, we reviewed the existing literature on quiescence and senescence in both human and mouse models [9, 19, 70–81], as detailed in the Additional file 3. We assessed the enrichment of several senescence-related gene sets, as well as two consensus gene sets we constructed, in DE results and in metaGEP signatures, indicating that the quiescence signal in our data can not be exclusively attributed to senescence (Additional file 3).

### Comparison with mouse HSC aging signature

To compare the aging signature produced in this study with a previously published mouse HSC aging [34], we extracted the mouse HSC aging signature from the Supplementary File of [34], which contained 179 genes positively associated with age in mice. We first identified the overlap between the mouse and human aging signatures by searching for orthologous genes. We identified seven genes shared between the two signatures: *CLU, NPDC1, JUN, EGR1, ZFP36, TNFSF10,* and *BTG2*. Among the 11,646 human genes present in the DE analysis and having a known mouse ortholog, 118 and 126 genes (adjusted *p*-value < 0.05, logFC > 0.5) belong to the mouse and human aging signatures, respectively, with an overlap of seven genes. This overlap is larger than expected by chance (Fisher’s Exact test *p*-value = 0.00029, odds ratio = 6.04). Interestingly, the mouse and human signatures share an AP-1 member (*JUN*), the anti-inflammatory gene *ZFP36*, and Clusterin (*CLU*) which was recently reported to mark a myeloid-biased population of mouse HSCs expanding with age [22]. We then conducted a gene set over-representation analysis on the mouse signature with *fgsea*, using HALLMARK and C2 collections of mouse signatures from MSigDB (Additional file 2: Fig. S14, Additional file 1: Table S4) [57–59].

## Supplementary Information

Additional file 1. Supplementary tables S1-S4.

Additional file 2. Supplementary figures S1-S15.

Additional file 3. Supplementary note exploring the signal of senescence.

## Data Availability

Sequencing data generated in this study were deposited to NCBI Gene Expression Omnibus under accession number GSE302126 [40]. Seurat objects necessary to reproduce the described analyses are available at Figshare [82]. Previously published datasets supporting the conclusions of this article are available at NCBI Gene Expression Omnibus, accession numbers GSE104408 [83], GSE180298 [84], GSE189161 [85], GSE120221 [86], and at http://scrna.sklehabc.com/HSPC/ [87]. Annotated code to reproduce all analyses is available on Github [88] and Zenodo [89] under MIT license.

## References

[1] MacKinney AA Jr. Effect of aging on the peripheral blood lymphocyte count. J Gerontol. 1978;33:213–6. 627705 10.1093/geronj/33.2.213

[2] Montecino-Rodriguez E, Berent-Maoz B, Dorshkind K. Causes, consequences, and reversal of immune system aging. J Clin Invest. 2013;123:958–65. 23454758 10.1172/JCI64096 PMC3582124

[3] Price EA. Aging and erythropoiesis: current state of knowledge. Blood Cells Mol Dis. 2008;41:158–65. 18514554 10.1016/j.bcmd.2008.04.005

[4] Weng C, Yu F, Yang D, Poeschla M, Liggett LA, Jones MG, et al. Deciphering cell states and genealogies of human haematopoiesis. Nature. 2024;627:389–98. 38253266 10.1038/s41586-024-07066-z PMC10937407

[5] Yamamoto R, Wilkinson AC, Ooehara J, Lan X, Lai CY, Nakauchi Y, et al. Large-scale clonal analysis resolves aging of the mouse hematopoietic stem cell compartment. Cell Stem Cell. 2018;22:600-7.e4. 29625072 10.1016/j.stem.2018.03.013 PMC5896201

[6] de Haan G, Lazare SS. Aging of hematopoietic stem cells. Blood. 2018;131(5):479–87. 29141947 10.1182/blood-2017-06-746412

[7] Leins H, Mulaw M, Eiwen K, Sakk V, Liang Y, Denkinger M, et al. Aged murine hematopoietic stem cells drive aging-associated immune remodeling. Blood. 2018;132:565–76. 29891535 10.1182/blood-2018-02-831065 PMC6137572

[8] Pang WW, Price EA, Sahoo D, Beerman I, Maloney WJ, Rossi DJ, et al. Human bone marrow hematopoietic stem cells are increased in frequency and myeloid-biased with age. Proc Natl Acad Sci USA. 2011;108:20012–7. 22123971 10.1073/pnas.1116110108 PMC3250139

[9] Amoah A, Keller A, Emini R, Hoenicka M, Liebold A, Vollmer A, et al. Aging of human hematopoietic stem cells is linked to changes in Cdc42 activity. Haematologica. 2022;107:393–402. 33440922 10.3324/haematol.2020.269670 PMC8804569

[10] Kuranda K, Vargaftig J, de la Rochere P, Dosquet C, Charron D, Bardin F, et al. Age-related changes in human hematopoietic stem/progenitor cells. Aging Cell. 2011;10:542–6. 21418508 10.1111/j.1474-9726.2011.00675.x

[11] Adelman ER, Huang HT, Roisman A, Olsson A, Colaprico A, Qin T, et al. Aging human hematopoietic stem cells manifest profound epigenetic reprogramming of enhancers that may predispose to leukemia. Cancer Discov. 2019;9:1080–101. 31085557 10.1158/2159-8290.CD-18-1474 PMC7080409

[12] Konturek-Ciesla A, Dhapola P, Zhang Q, Säwén P, Wan H, Karlsson G, et al. Temporal multimodal single-cell profiling of native hematopoiesis illuminates altered differentiation trajectories with age. Cell Rep. 2023;42:112304. 36961818 10.1016/j.celrep.2023.112304

[13] Zeng AGX, Nagree MS, Jakobsen NA, Shah S, Murison A, Cheong JG, et al. Identification of a human hematopoietic stem cell subset that retains memory of inflammatory stress. bioRxiv. 2023:2023–09. 10.1101/2023.09.11.557271.

[14] Jakobsen NA, Turkalj S, Zeng AGX, Stoilova B, Metzner M, Rahmig S, et al. Selective advantage of mutant stem cells in human clonal hematopoiesis is associated with attenuated response to inflammation and aging. Cell Stem Cell. 2024;31:1127-44.e17. 38917807 10.1016/j.stem.2024.05.010 PMC11512683

[15] Santaguida M, Schepers K, King B, Sabnis AJ, Forsberg EC, Attema JL, et al. JunB protects against myeloid malignancies by limiting hematopoietic stem cell proliferation and differentiation without affecting self-renewal. Cancer Cell. 2009;15:341–52. 19345332 10.1016/j.ccr.2009.02.016 PMC2669108

[16] Okada S, Fukuda T, Inada K, Tokuhisa T. Prolonged expression of c-fos suppresses cell cycle entry of dormant hematopoietic stem cells. Blood. 1999;93:816–25. 9920830

[17] Wagner EF, Nebreda AR. Signal integration by JNK and p38 MAPK pathways in cancer development. Nat Rev Cancer. 2009;9:537–49. 19629069 10.1038/nrc2694

[18] Alvarez-Rodríguez L, López-Hoyos M, Muñoz-Cacho P, Martínez-Taboada VM. Aging is associated with circulating cytokine dysregulation. Cell Immunol. 2012;273:124–32. 22316526 10.1016/j.cellimm.2012.01.001

[19] Noda S, Ichikawa H, Miyoshi H. Hematopoietic stem cell aging is associated with functional decline and delayed cell cycle progression. Biochem Biophys Res Commun. 2009;383:210–5. 19345668 10.1016/j.bbrc.2009.03.153

[20] Kotliar D, Veres A, Nagy MA, Tabrizi S, Hodis E, Melton DA, et al. Identifying gene expression programs of cell-type identity and cellular activity with single-cell RNA-Seq. Elife. 2019;8:e43803. 31282856 10.7554/eLife.43803 PMC6639075

[21] Henry CJ, Marusyk A, Zaberezhnyy V, Adane B, DeGregori J. Declining lymphoid progenitor fitness promotes aging-associated leukemogenesis. Proc Natl Acad Sci U S A. 2010;107:21713–8. 21098275 10.1073/pnas.1005486107 PMC3003039

[22] Min IM, Pietramaggiori G, Kim FS, Passegué E, Stevenson KE, Wagers AJ. The transcription factor EGR1 controls both the proliferation and localization of hematopoietic stem cells. Cell Stem Cell. 2008;2:380–91. 18397757 10.1016/j.stem.2008.01.015

[23] Park CS, Bridges CS, Lewis AH, Chen TJ, Shai S, Du W, et al. *KLF4* enhances transplantation-induced hematopoiesis by inhibiting *TLRs* and noncanonical *NFκB* signaling at a steady state. Exp Hematol. 2025;144:104730. 39900173 10.1016/j.exphem.2025.104730 PMC12261498

[24] Davis RJ. Signal transduction by the JNK group of MAP kinases. Cell. 2000;103:239–52. 11057897 10.1016/s0092-8674(00)00116-1

[25] Konturek-Ciesla A, Olofzon R, Kharazi S, Bryder D. Implications of stress-induced gene expression for hematopoietic stem cell aging studies. Nat Aging. 2024;4:177–84. 38228925 10.1038/s43587-023-00558-z PMC10878961

[26] van den Brink SC, Sage F, Vértesy Á, Spanjaard B, Peterson-Maduro J, Baron CS, et al. Single-cell sequencing reveals dissociation-induced gene expression in tissue subpopulations. Nat Methods. 2017;14:935–6. 28960196 10.1038/nmeth.4437

[27] Essers MAG, Offner S, Blanco-Bose WE, Waibler Z, Kalinke U, Duchosal MA, et al. IFNalpha activates dormant haematopoietic stem cells in vivo. Nature. 2009;458:904–8. 19212321 10.1038/nature07815

[28] Baldridge MT, King KY, Boles NC, Weksberg DC, Goodell MA. Quiescent haematopoietic stem cells are activated by IFN-gamma in response to chronic infection. Nature. 2010;465:793–7. 20535209 10.1038/nature09135 PMC2935898

[29] Bogeska R, Mikecin AM, Kaschutnig P, Fawaz M, Büchler-Schäff M, Le D, et al. Inflammatory exposure drives long-lived impairment of hematopoietic stem cell self-renewal activity and accelerated aging. Cell Stem Cell. 2022;29:1273-84.e8. 35858618 10.1016/j.stem.2022.06.012 PMC9357150

[30] Chavez JS, Rabe JL, Loeffler D, Higa KC, Hernandez G, Mills TS, et al. PU.1 enforces quiescence and limits hematopoietic stem cell expansion during inflammatory stress. J Exp Med. 2021;218(6):e20201169. 33857288 10.1084/jem.20201169 PMC8056754

[31] Pietras EM, Lakshminarasimhan R, Techner J-M, Fong S, Flach J, Binnewies M, et al. Re-entry into quiescence protects hematopoietic stem cells from the killing effect of chronic exposure to type I interferons. J Exp Med. 2014;211:245–62. 24493802 10.1084/jem.20131043 PMC3920566

[32] Kain BN, Tran BT, Luna PN, Cao R, Le DT, Florez MA, et al. Hematopoietic stem and progenitor cells confer cross-protective trained immunity in mouse models. iScience. 2023;26:107596. 37664586 10.1016/j.isci.2023.107596 PMC10470378

[33] Cheong JG, Ravishankar A, Sharma S, Parkhurst CN, Grassmann SA, Wingert CK, et al. Epigenetic memory of coronavirus infection in innate immune cells and their progenitors. Cell. 2023;186:3882-902.e24. 37597510 10.1016/j.cell.2023.07.019 PMC10638861

[34] Flohr Svendsen A, Yang D, Kim K, Lazare S, Skinder N, Zwart E, et al. A comprehensive transcriptome signature of murine hematopoietic stem cell aging. Blood. 2021;138:439–51. 33876187 10.1182/blood.2020009729

[35] Cabezas-Wallscheid N, Buettner F, Sommerkamp P, Klimmeck D, Ladel L, Thalheimer FB, et al. Vitamin A-retinoic acid signaling regulates hematopoietic stem cell dormancy. Cell. 2017;169:807-23.e19. 28479188 10.1016/j.cell.2017.04.018

[36] Sousa-Victor P, Gutarra S, García-Prat L, Rodriguez-Ubreva J, Ortet L, Ruiz-Bonilla V, et al. Geriatric muscle stem cells switch reversible quiescence into senescence. Nature. 2014;506:316–21. 24522534 10.1038/nature13013

[37] Patrick R, Naval-Sanchez M, Deshpande N, Huang Y, Zhang J, Chen X, et al. The activity of early-life gene regulatory elements is hijacked in aging through pervasive AP-1-linked chromatin opening. Cell Metab. 2024;36:1858-81.e23. 38959897 10.1016/j.cmet.2024.06.006

[38] Jacobs MD, Harrison SC. Structure of an IkappaBalpha/NF-kappaB complex. Cell. 1998;95:749–58. 9865693 10.1016/s0092-8674(00)81698-0

[39] Tanaka-Yano M, Zong L, Park B, Yanai H, Tekin-Turhan F, Blackshear PJ, et al. Tristetraprolin overexpression drives hematopoietic changes in young and middle-aged mice generating dominant mitigating effects on induced inflammation in murine models. GeroScience. 2024;46:1271–84. 37535204 10.1007/s11357-023-00879-2 PMC10828162

[40] Safina KR, Good JD, van Galen P. An inflammatory and quiescent HSC subpopulation expands with age in humans. NCBI Gene Expression Omnibus. 2025. https://www.ncbi.nlm.nih.gov/geo/query/acc.cgi?acc=GSE302126. 10.1186/s13059-026-03936-z PMC12892447 41546063

[41] Ainciburu M, Ezponda T, Berastegui N, Alfonso-Pierola A, Vilas-Zornoza A, San Martin-Uriz P, et al. Uncovering perturbations in human hematopoiesis associated with healthy aging and myeloid malignancies at single-cell resolution. Elife. 2023;12:e79363. 36629404 10.7554/eLife.79363 PMC9904760

[42] Li H, Côté P, Kuoch M, Ezike J, Frenis K, Afanassiev A, et al. The dynamics of hematopoiesis over the human lifespan. Nat Methods. 2025;22:422–34. 39639169 10.1038/s41592-024-02495-0 PMC11908799

[43] Oetjen KA, Lindblad KE, Goswami M, Gui G, Dagur PK, Lai C, et al. Human bone marrow assessment by single-cell RNA sequencing, mass cytometry, and flow cytometry. JCI Insight. 2018;3:e124928. 30518681 10.1172/jci.insight.124928 PMC6328018

[44] Zhang Y, Xie X, Huang Y, Liu M, Li Q, Luo J, et al. Temporal molecular program of human hematopoietic stem and progenitor cells after birth. Dev Cell. 2022;57:2745-60.e6. 36493772 10.1016/j.devcel.2022.11.013

[45] Dobin A, Davis CA, Schlesinger F, Drenkow J, Zaleski C, Jha S, et al. STAR: ultrafast universal RNA-seq aligner. Bioinformatics. 2013;29:15–21. 23104886 10.1093/bioinformatics/bts635 PMC3530905

[46] Liao Y, Smyth GK, Shi W. The R package Rsubread is easier, faster, cheaper and better for alignment and quantification of RNA sequencing reads. Nucleic Acids Res. 2019;47:e47. 30783653 10.1093/nar/gkz114 PMC6486549

[47] Hao Y, Stuart T, Kowalski MH, Choudhary S, Hoffman P, Hartman A, et al. Dictionary learning for integrative, multimodal and scalable single-cell analysis. Nat Biotechnol. 2024;42:293–304. 37231261 10.1038/s41587-023-01767-y PMC10928517

[48] GitHub - Moonerss/scrubletR. GitHub. https://github.com/Moonerss/scrubletR.

[49] Zeng AGX, Iacobucci I, Shah S, Mitchell A, Wong G, Bansal S, et al. Single-cell transcriptional atlas of human hematopoiesis reveals genetic and hierarchy-based determinants of aberrant AML differentiation. Blood Cancer Discov. 2025;6:307–24. 40294241 10.1158/2643-3230.BCD-24-0342 PMC12209776

[50] Gayoso A, Lopez R, Xing G, Boyeau P, Valiollah Pour Amiri V, Hong J, et al. A python library for probabilistic analysis of single-cell omics data. Nat Biotechnol. 2022;40:163–6. 35132262 10.1038/s41587-021-01206-w

[51] Laurenti E, Doulatov S, Zandi S, Plumb I, Chen J, April C, et al. The transcriptional architecture of early human hematopoiesis identifies multilevel control of lymphoid commitment. Nat Immunol. 2013;14:756–63. 23708252 10.1038/ni.2615 PMC4961471

[52] Eppert K, Takenaka K, Lechman ER, Waldron L, Nilsson B, van Galen P, et al. Stem cell gene expression programs influence clinical outcome in human leukemia. Nat Med. 2011;17:1086–93. 21873988 10.1038/nm.2415

[53] Jaatinen T, Hemmoranta H, Hautaniemi S, Niemi J, Nicorici D, Laine J, et al. Global gene expression profile of human cord blood-derived CD133+ cells. Stem Cells. 2006;24:631–41. 16210406 10.1634/stemcells.2005-0185

[54] Love MI, Huber W, Anders S. Moderated estimation of fold change and dispersion for RNA-seq data with DESeq2. Genome Biol. 2014;15:550. 25516281 10.1186/s13059-014-0550-8 PMC4302049

[55] Zhu A, Ibrahim JG, Love MI. Heavy-tailed prior distributions for sequence count data: removing the noise and preserving large differences. Bioinformatics. 2019;35:2084–92. 30395178 10.1093/bioinformatics/bty895 PMC6581436

[56] Korotkevich G, Sukhov V, Budin N, Shpak B, Artyomov MN, Sergushichev A. Fast gene set enrichment analysis. bioRxiv. 2021:060012. 10.1101/060012.

[57] Subramanian A, Tamayo P, Mootha VK, Mukherjee S, Ebert BL, Gillette MA, et al. Gene set enrichment analysis: a knowledge-based approach for interpreting genome-wide expression profiles. Proc Natl Acad Sci U S A. 2005;102:15545–50. 16199517 10.1073/pnas.0506580102 PMC1239896

[58] Liberzon A, Subramanian A, Pinchback R, Thorvaldsdóttir H, Tamayo P, Mesirov JP. Molecular signatures database (MSigDB) 3.0. Bioinformatics. 2011;27:1739–40. 21546393 10.1093/bioinformatics/btr260 PMC3106198

[59] Liberzon A, Birger C, Thorvaldsdóttir H, Ghandi M, Mesirov JP, Tamayo P. The molecular signatures database (MSigDB) hallmark gene set collection. Cell Syst. 2015;1:417–25. 26771021 10.1016/j.cels.2015.12.004 PMC4707969

[60] García-Prat L, Kaufmann KB, Schneiter F, Voisin V, Murison A, Chen J, et al. TFEB-mediated endolysosomal activity controls human hematopoietic stem cell fate. Cell Stem Cell. 2021;28:1838-50.e10. 34343492 10.1016/j.stem.2021.07.003

[61] Rundberg Nilsson A, Soneji S, Adolfsson S, Bryder D, Pronk CJ. Human and murine hematopoietic stem cell aging is associated with functional impairments and intrinsic megakaryocytic/erythroid bias. PLoS One. 2016;11:e0158369. 27368054 10.1371/journal.pone.0158369 PMC4930192

[62] Hennrich ML, Romanov N, Horn P, Jaeger S, Eckstein V, Steeples V, et al. Cell-specific proteome analyses of human bone marrow reveal molecular features of age-dependent functional decline. Nat Commun. 2018;9:4004. 30275468 10.1038/s41467-018-06353-4 PMC6167374

[63] Gavish A, Tyler M, Greenwald AC, Hoefflin R, Simkin D, Tschernichovsky R, et al. Hallmarks of transcriptional intratumour heterogeneity across a thousand tumours. Nature. 2023;618:598–606. 37258682 10.1038/s41586-023-06130-4

[64] Kotliar D, Curtis M, Agnew R, Weinand K, Nathan A, Baglaenko Y, et al. Reproducible single-cell annotation of programs underlying T cell subsets, activation states and functions. Nat Methods. 2025;22:1964–80. 40903640 10.1038/s41592-025-02793-1 PMC12446064

[65] Aibar S, González-Blas CB, Moerman T, Huynh-Thu VA, Imrichova H, Hulselmans G, et al. SCENIC: single-cell regulatory network inference and clustering. Nat Methods. 2017;14:1083–6. 28991892 10.1038/nmeth.4463 PMC5937676

[66] Reshef YA, Rumker L, Kang JB, Nathan A, Korsunsky I, Asgari S, et al. Co-varying neighborhood analysis identifies cell populations associated with phenotypes of interest from single-cell transcriptomics. Nat Biotechnol. 2022;40:355–63. 34675423 10.1038/s41587-021-01066-4 PMC8930733

[67] Takase S, Lerond L, Bergan JJ, Schmid-Schönbein GW. The inflammatory reaction during venous hypertension in the rat. Microcirculation. 2000;7:41–52. 10708336

[68] Zou J, Yuan D, Yang J, Yu Y. Effects of diosmin on vascular leakage and inflammation in a mouse model of venous obstruction. Front Nutr. 2022;9:831485. 35273990 10.3389/fnut.2022.831485 PMC8903897

[69] Rettkowski J, Romero-Mulero MC, Singh I, Wadle C, Wrobel J, Chiang D, et al. Modulation of bone marrow haematopoietic stem cell activity as a therapeutic strategy after myocardial infarction: a preclinical study. Nat Cell Biol. 2025;27:591–604. 40175666 10.1038/s41556-025-01639-4 PMC11991920

[70] Flach J, Bakker ST, Mohrin M, Conroy PC, Pietras EM, Reynaud D, et al. Replication stress is a potent driver of functional decline in ageing haematopoietic stem cells. Nature. 2014;512:198–202. 25079315 10.1038/nature13619 PMC4456040

[71] Kirschner K, Chandra T, Kiselev V, Flores-Santa Cruz D, Macaulay IC, Park HJ, et al. Proliferation drives aging-related functional decline in a subpopulation of the hematopoietic stem cell compartment. Cell Rep. 2017;19:1503–11. 28538171 10.1016/j.celrep.2017.04.074 PMC5457484

[72] Kovtonyuk LV, Ashcroft P, Spaltro G, Tata NR, Skoda RC, Bonhoeffer S, et al. Hematopoietic stem cells increase quiescence during aging. Blood. 2019;134:2484–2484.

[73] Su TY, Hauenstein J, Somuncular E, Dumral Ö, Leonard E, Gustafsson C, et al. Aging is associated with functional and molecular changes in distinct hematopoietic stem cell subsets. Nat Commun. 2024;15:1–16. 39261515 10.1038/s41467-024-52318-1 PMC11391069

[74] Poisa-Beiro L, Landry JJM, Yan B, Kardorff M, Eckstein V, Villacorta L, et al. A senescent cluster in aged human hematopoietic stem cell compartment as target for senotherapy. Int J Mol Sci. 2025;26:787. 39859500 10.3390/ijms26020787 PMC11766015

[75] Suryadevara V, Hudgins AD, Rajesh A, Pappalardo A, Karpova A, Dey AK, et al. Sennet recommendations for detecting senescent cells in different tissues. Nat Rev Mol Cell Biol. 2024;25:1001–23. 38831121 10.1038/s41580-024-00738-8 PMC11578798

[76] Reimann M, Lee S, Schmitt CA. Cellular senescence: neither irreversible nor reversible. J Exp Med. 2024;221:e20232136. 38385946 10.1084/jem.20232136 PMC10883852

[77] Ashraf HM, Fernandez B, Spencer SL. The intensities of canonical senescence biomarkers integrate the duration of cell-cycle withdrawal. Nat Commun. 2023;14:4527. 37500655 10.1038/s41467-023-40132-0 PMC10374620

[78] Wang J, Sun Q, Morita Y, Jiang H, Groß A, Lechel A, et al. A differentiation checkpoint limits hematopoietic stem cell self-renewal in response to DNA damage. Cell. 2014;158:1444. 28915370 10.1016/j.cell.2014.08.033

[79] Cheng T, Rodrigues N, Shen H, Yang Y, Dombkowski D, Sykes M, et al. Hematopoietic stem cell quiescence maintained by p21cip1/waf1. Science. 2000;287:1804–8. 10710306 10.1126/science.287.5459.1804

[80] Perucca P, Cazzalini O, Madine M, Savio M, Laskey RA, Vannini V, et al. Loss of p21 CDKN1A impairs entry to quiescence and activates a DNA damage response in normal fibroblasts induced to quiescence. Cell Cycle. 2009;8:105–14. 19106607 10.4161/cc.8.1.7507

[81] Saul D, Kosinsky RL, Atkinson EJ, Doolittle ML, Zhang X, LeBrasseur NK, et al. A new gene set identifies senescent cells and predicts senescence-associated pathways across tissues. Nat Commun. 2022;13:4827. 35974106 10.1038/s41467-022-32552-1 PMC9381717

[82] Safina KR, van Galen P. Aging_HSCs_2025. Figshare. 2025. https://figshare.com/projects/Aging_HSCs_2025/235781.

[83] Adelman ER, Huang HT, Roisman A, Olsson A et al. Aging human hematopoietic stem cells manifest profound epigenetic reprogramming of enhancers that may predispose to leukemia. NCBI Gene Expression Omnibus. 2019. https://www.ncbi.nlm.nih.gov/geo/query/acc.cgi?acc=GSE104408. 10.1158/2159-8290.CD-18-1474 PMC7080409 31085557

[84] Ainciburu M, Ezponda T, Berastegui N, Alfonso-Pierola A, Vilas-Zornoza A, San Martin-Uriz P, et al. Uncovering perturbations in human hematopoiesis associated with healthy aging and myeloid malignancies at single cell resolution. NCBI Gene Expression Omnibus. 2021. https://www.ncbi.nlm.nih.gov/geo/query/acc.cgi?acc=GSE180298. 10.7554/eLife.79363 PMC9904760 36629404

[85] Li H, Butty VL, Kuoch M, Cote P, Chou ST, Rowe RG. Hematopoiesis at single cell resolution spanning human development and maturation. NCBI Gene Expression Omnibus. 2024. https://www.ncbi.nlm.nih.gov/geo/query/acc.cgi?acc=GSE189161.

[86] Oetjen KA, Gui G, Hourigan CS. Human bone marrow assessment by single cell RNA sequencing, mass cytometry and flow cytometry [scRNA]. NCBI Gene Expression Omnibus. 2018. https://www.ncbi.nlm.nih.gov/geo/query/acc.cgi?acc=GSE120221. 10.1172/jci.insight.124928 PMC6328018 30518681

[87] Zhang Y, Xie X, Huang Y, Liu M, Li Q, Luo J, et al. Atlas of human HSPCs after birth. 2022. http://scrna.sklehabc.com/HSPC/.

[88] Safina KR. An inflammatory and quiescent HSC subpopulation expands with age in humans. Github. 2025. https://github.com/noranekonobokkusu/Aging_HSCs_2025. 10.1186/s13059-026-03936-z PMC12892447 41546063

[89] Safina KR, van Galen P. An inflammatory and quiescent HSC subpopulation expands with age in humans. 2025. Zenodo. 10.5281/zenodo.17992640. 10.1186/s13059-026-03936-z PMC12892447 41546063

