# Supplementary material for: An inflammatory and quiescent HSC subpopulation expands with age in humans
Source: Genome Biol. 2026 Jan 16;27:30. doi: 10.1186/s13059-026-03936-z (PMC12892447; doi:10.1186/s13059-026-03936-z)
Supplement: Supplementary file 2 — Additional file 2. Supplementary figures S1-S15. [file 13059_2026_3936_MOESM2_ESM.pdf]

Supplementary Figures

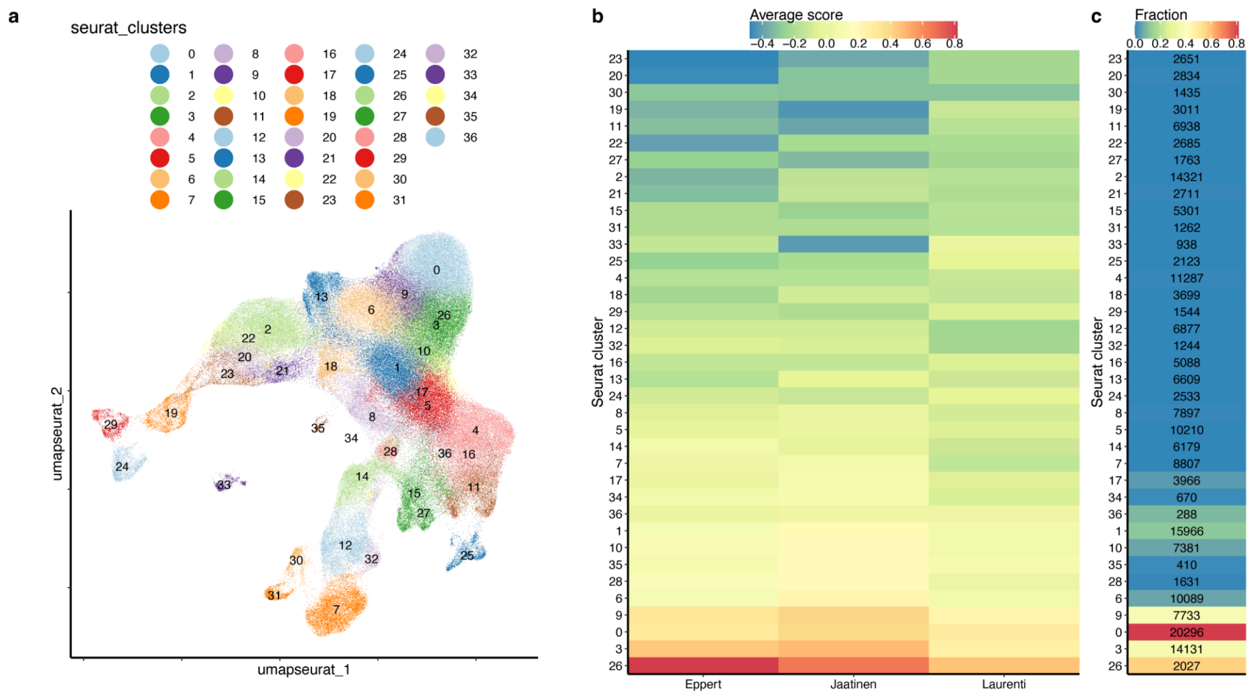

Fig. S1. Orthogonal HSC annotation agrees with BoneMarrowMap- produced HSC annotation. a. UMAP shows Louvain clusters identified on the integrated HSPC object. **b.** Heatmap shows the average module scores of three HSC-related public signatures across the Louvain clusters; clusters are sorted by the average score across the three signatures. **c.** Heatmap shows the fraction of cells annotated as HSCs by BMM per cluster; text shows the total number of cells per cluster. Clusters 0, 9, 3, and 26 sum up to 44,187 cells which includes 87% of the 28,989 BoneMarrowMap-annotated HSCs.

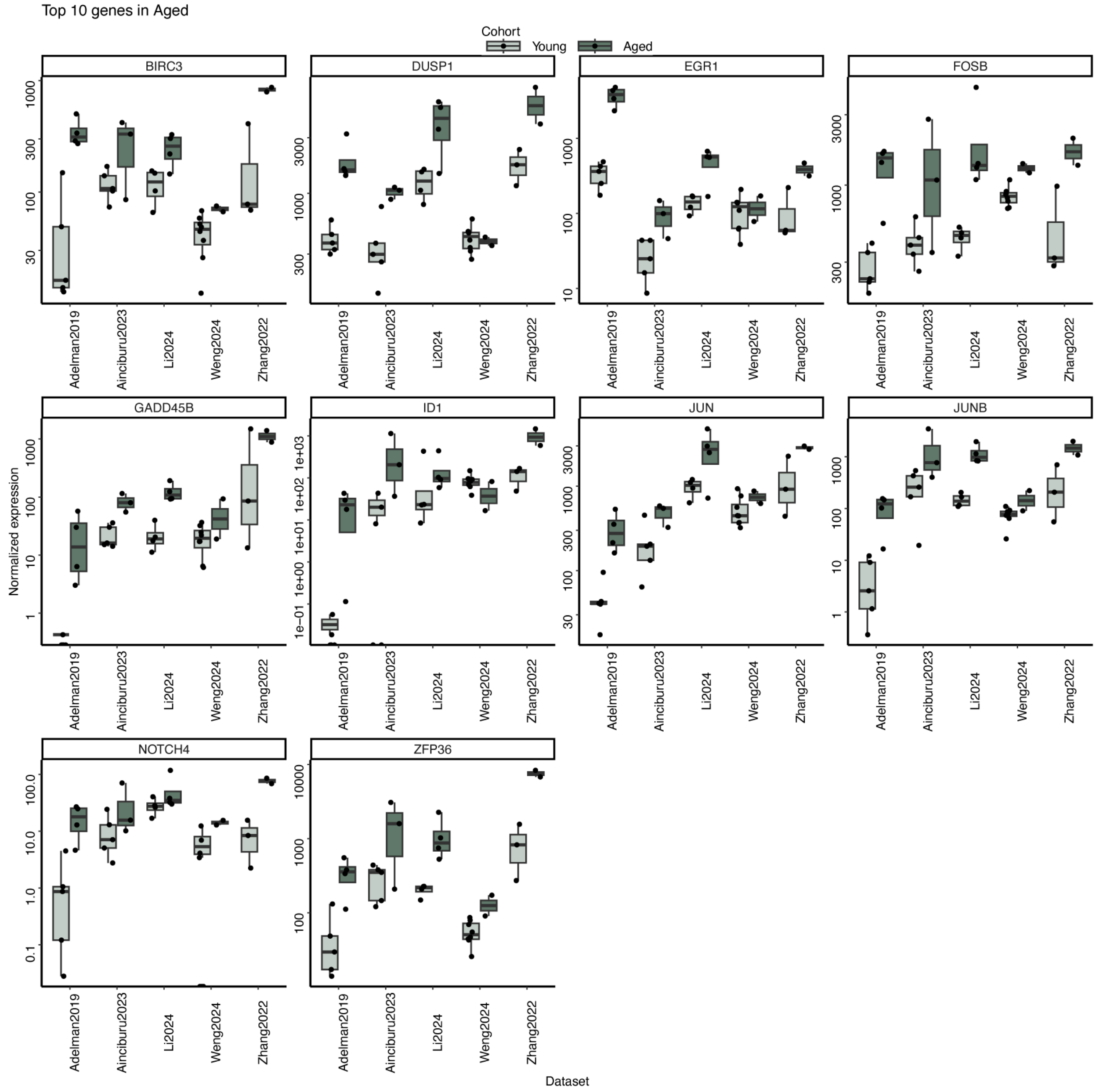

Fig. S2. Differential gene expression analysis reveals age-associated expression changes. Box plots show normalized expression of top-10 genes most significantly up-regulated in the Aged cohort across five datasets used for DE analysis. Each symbol represents an individual donor sample.

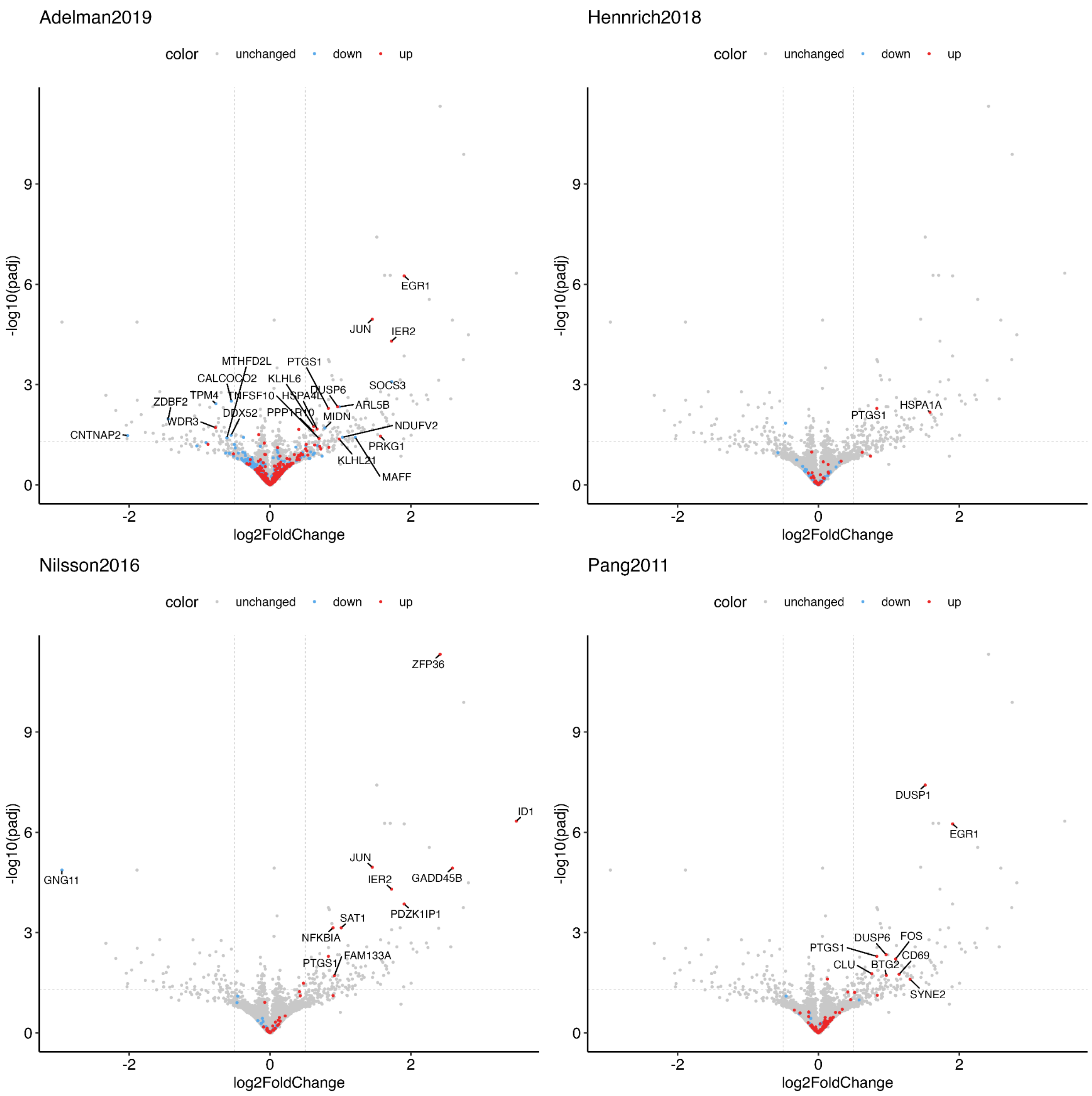

Fig. S3. Volcano plot of the DE results of this study shows the overlap with age-associated signatures from prior bulk RNA-seq studies. Positive log fold change indicates upregulation with age. Red and blue dots mark genes up- or down-regulated with age as identified in the prior study, so that genes with the direction of the effect concordant between this and the prior study are shown blue on the left and red on the right. Genes that show significant de-regulation in this study are labeled.

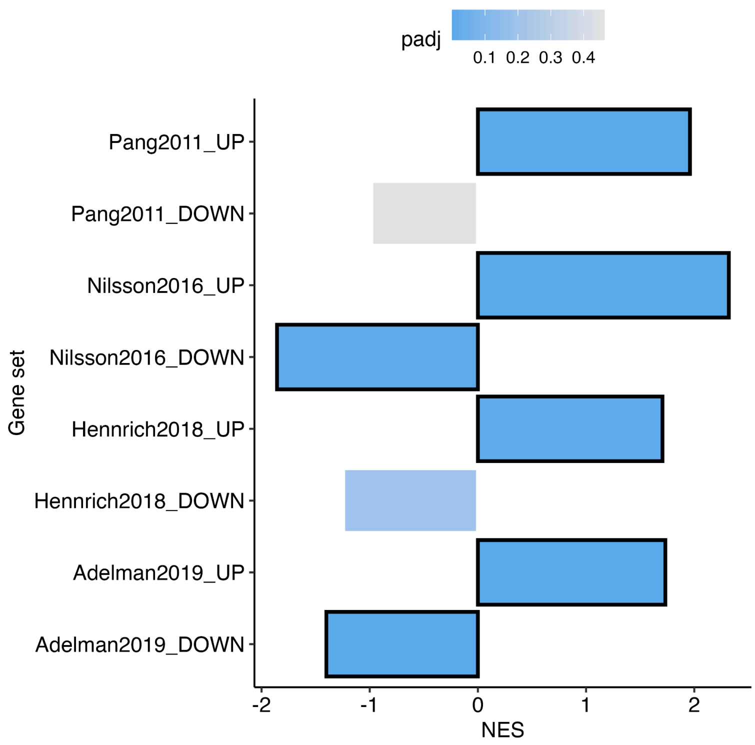

Fig. S4. GSEA reveals that genes identified as positively associated with age in prior studies are enriched among genes upregulated with age in our analysis. Gene sets with significant (adjusted p-value <0.05) enrichment are marked with black contour. NES, normalized enrichment score.

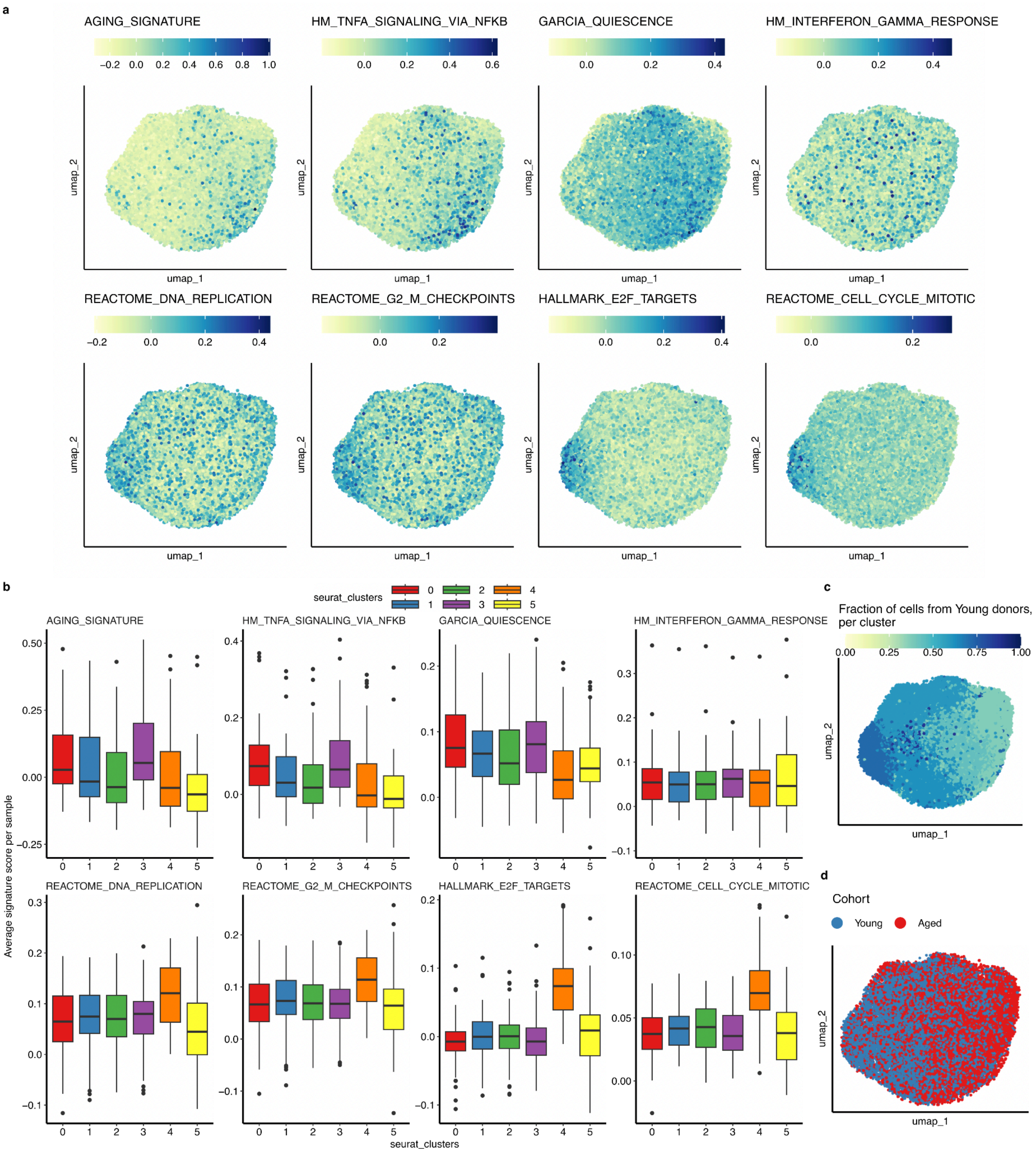

Fig. S5. The population of HSCs is not uniform. a. UMAP shows the activity of representative gene sets up-regulated in Aged (top row) and Young (bottom row) samples**. b.** Boxplot shows average signature scores of gene sets in **a** per sample across Seurat clusters. Only samples with at least 20 cells are used. **c**. UMAP colored by the fraction of cells coming from Young

donors per cluster. Note that the contribution of the Young cohort is higher in the left part of the UMAP. **d**. UMAP colored by age cohort. Only young and aged samples are shown.

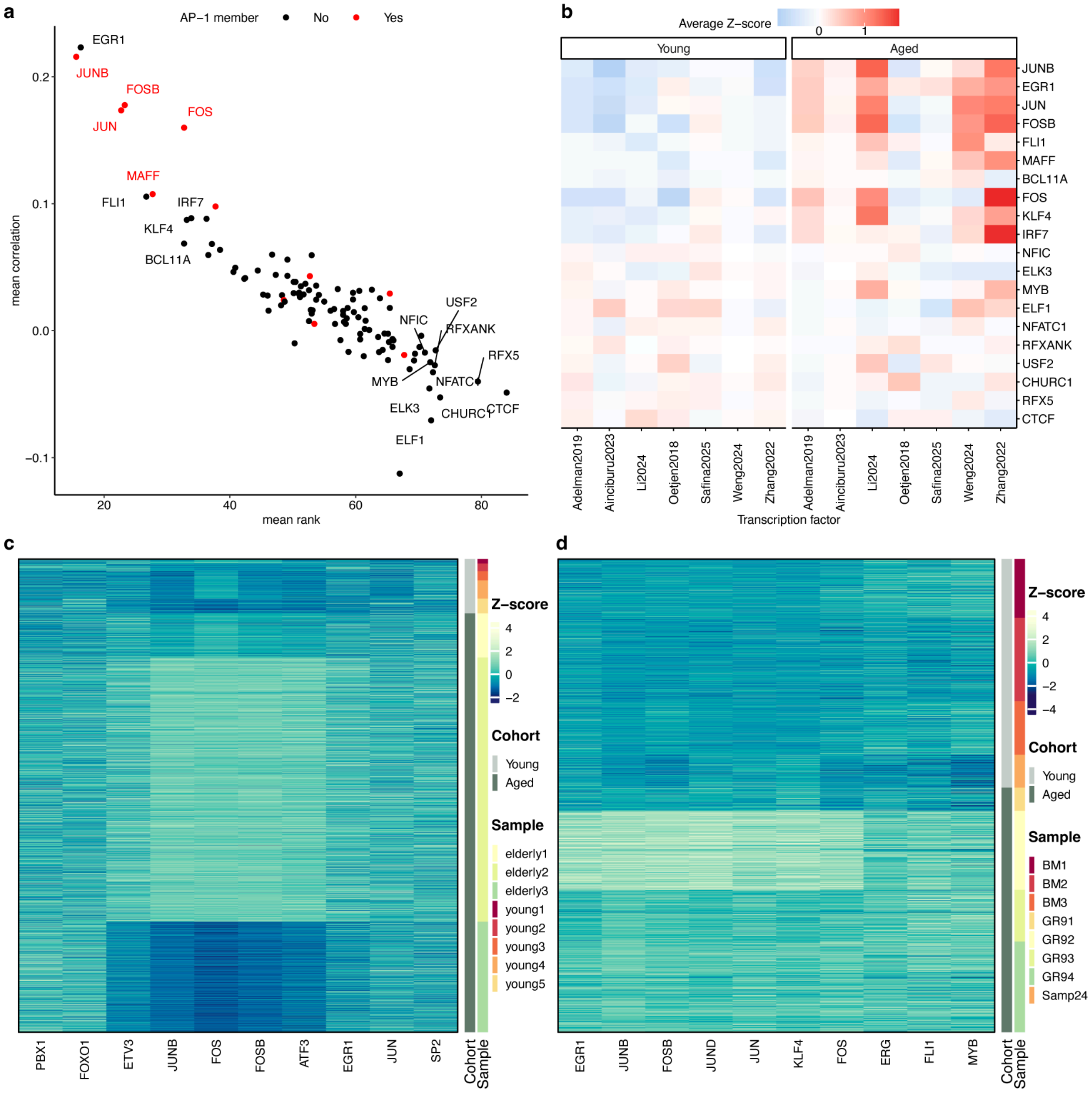

Fig. S6. Combined analysis of age-associated transcription factor activities. a. Scatter plot shows the mean ranks (x) vs. mean correlation (y) with age status of 108 TFs recovered in SCENIC analysis. Top-10 and bottom-10 TFs are labeled, AP-1 members are marked red. **b.** Heatmap shows the average Z-score of TFs labeled in (a) across all datasets. **c, d.** Heatmaps show the activity of top-10 age-associated TFs (columns) in cells (rows) from two example

datasets: Ainciburu (c) and Li (d). Significant variability between samples can be observed, including samples within the same age group.

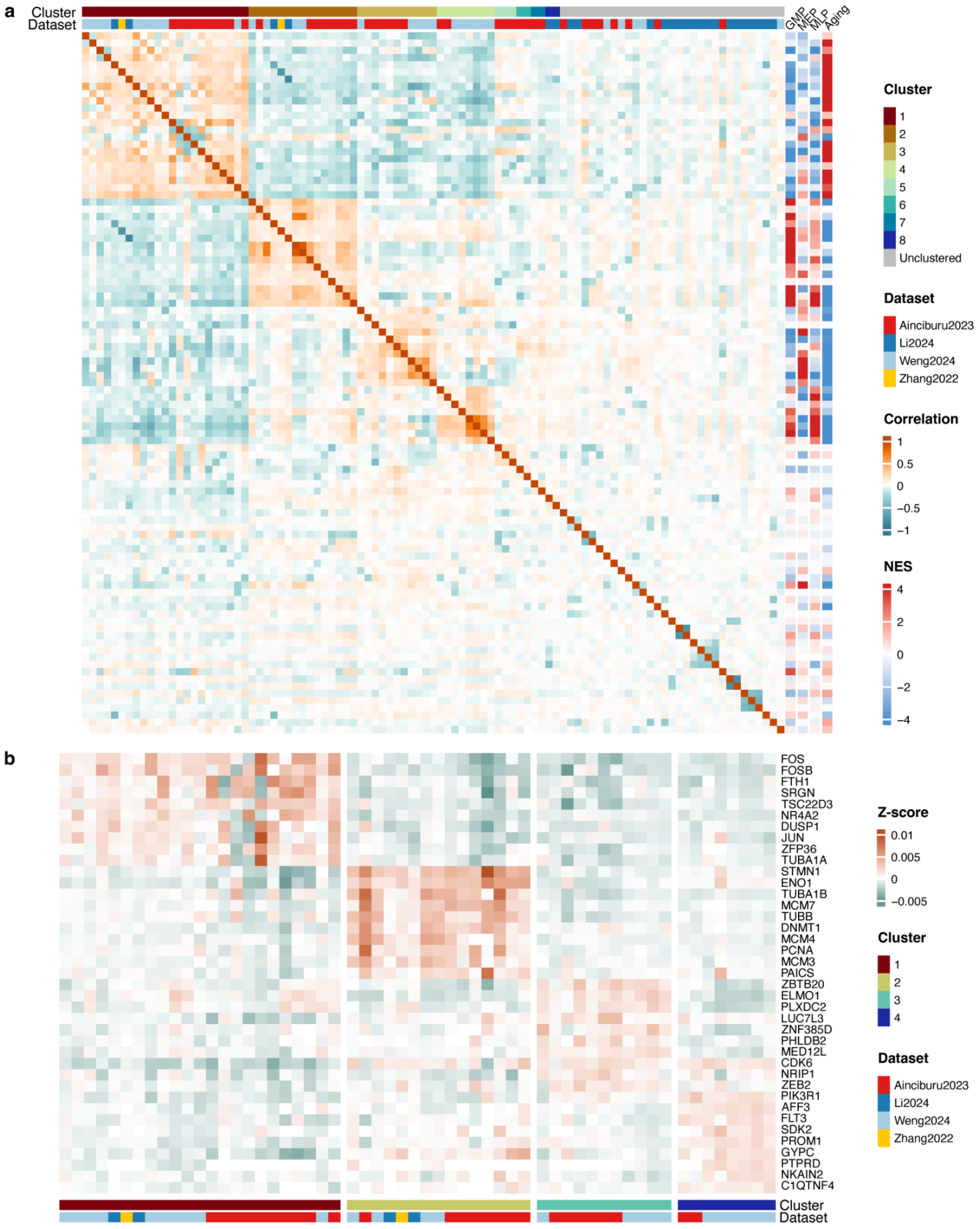

Fig. S7. cNMF analysis infers four metaGEPs. a. Heatmap shows clustering of all 97 programs discovered across 22 samples. This is an extended version of Fig. 2b. The side bars

show NES for characteristic signatures, indicating consistent enrichment of signatures across multiple programs within clusters. **b.** Heatmap shows Z-scores for top-10 genes associated with each cluster (metaGEP), highlighting consistent enrichment across most programs within each cluster.

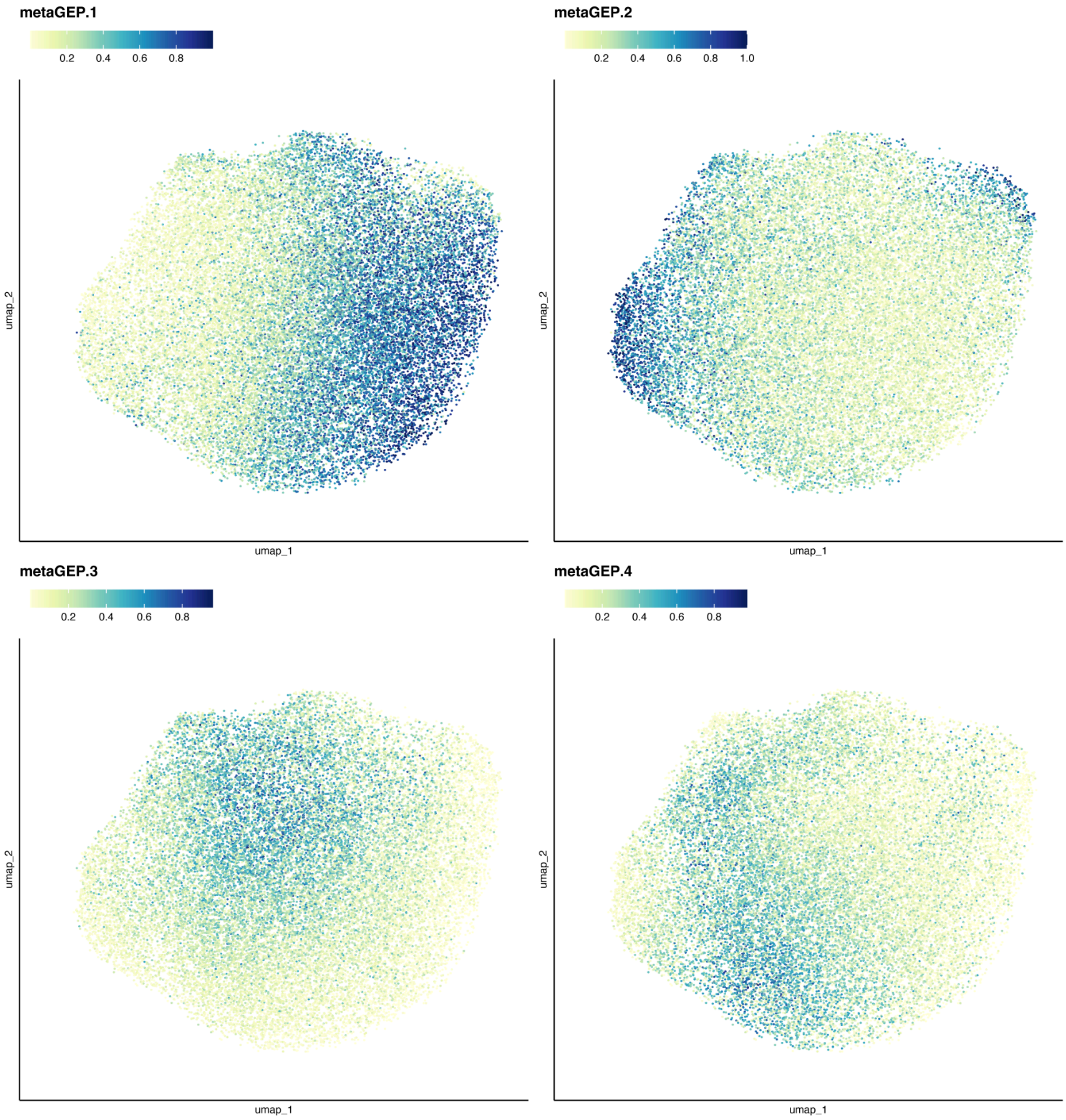

Fig. S8. Activities of metaGEPs are not uniform across HSCs. UMAPs are colored by usages of metaGEPs 1-4, normalized to a total of one by starCAT.

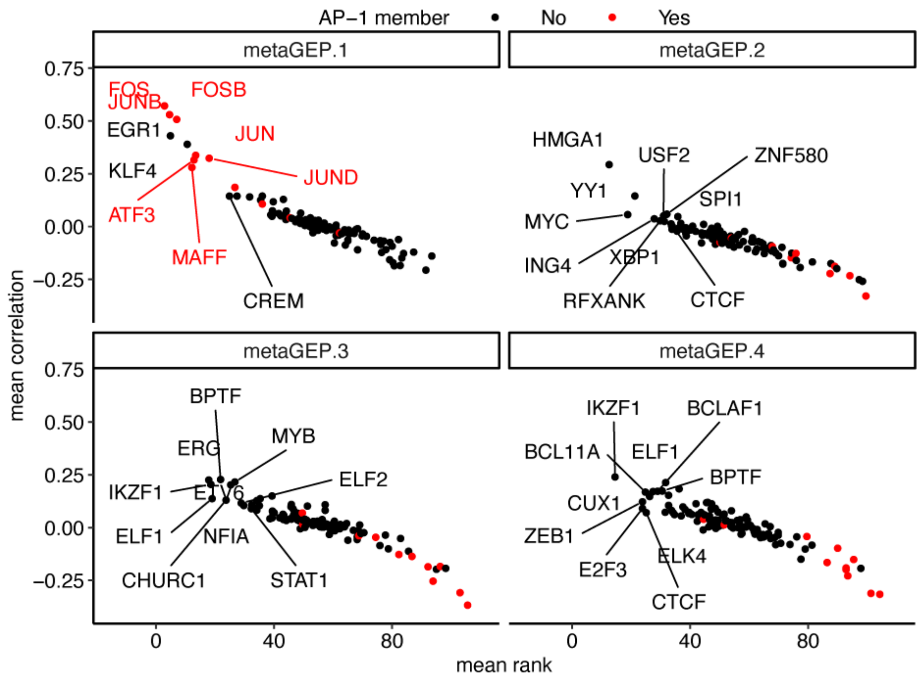

Fig. S9. metaGEPs are characterized by specific TF activities. Scatter plots show TFs (symbols) by their average rank across seven datasets (x) vs. their average correlation of AUC scores and metaGEP usages (y). Top-10 TFs per metaGEP are labeled; AP-1 members are shown in red. TFs with mean rank <=10 and mean correlation >=0.15 were selected for visualization in **Fig. 2f**. Compared to metaGEP1, correlation of TF activities with metaGEPs 2-4 was weaker, revealing overlapping TFs, particularly between metaGEPs 3-4. Thus, in contrast to the easily recoverable inflammatory aging program, metaGEP1, lineage priming programs may require more data to be reliably inferred and disentangled at the level of TF activity.

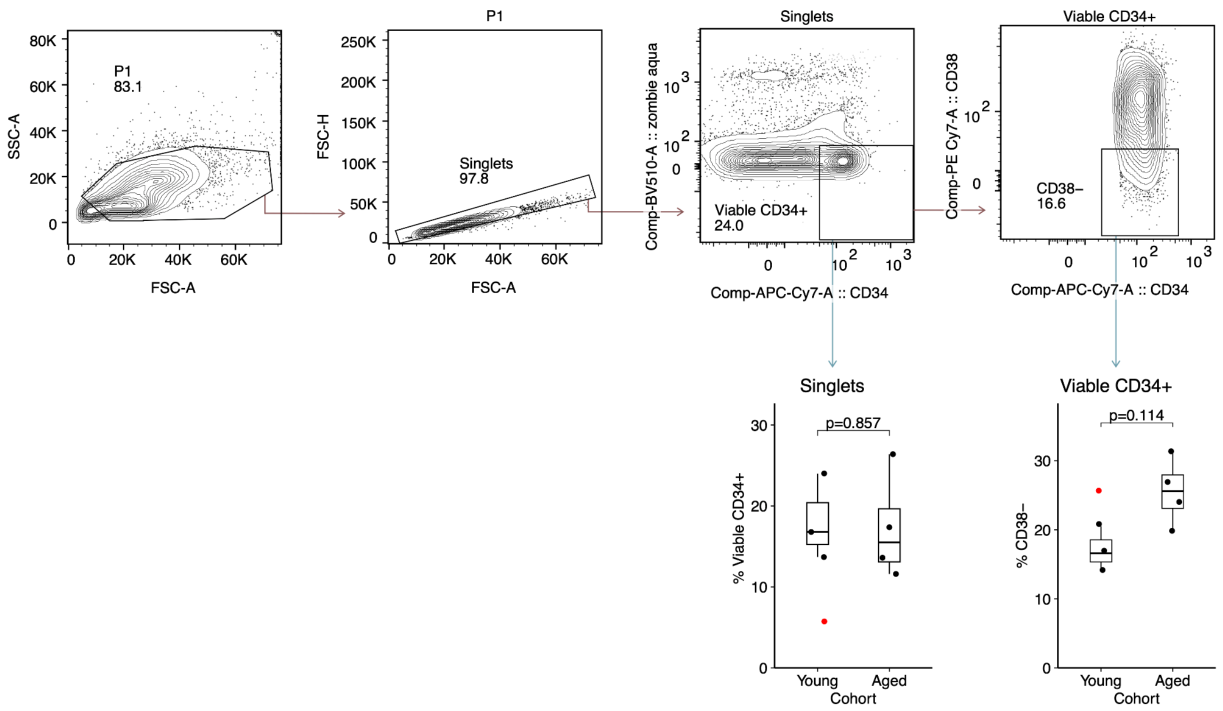

Fig. S10. Gating strategy. Percentages of gated populations are shown for a representative sample (SBM1001). Boxplots show percentages for eight analyzed samples. P-values were calculated using the paired Wilcoxon test. The red dot marks an outlier sample(SBM1148), which was excluded from statistical analyses.

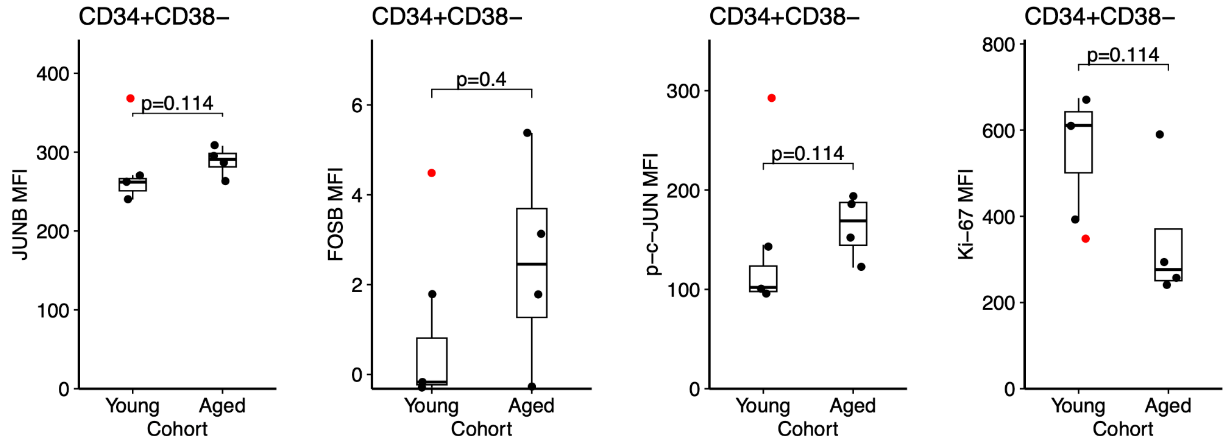

Fig. S11. MFIs of JUNB, FOSB, p-c-JUN and Ki-67 across CD34+CD48- populations in young and aged cohorts. Boxplots show MFI (mean fluorescent intensities) of four intracellular proteins. P-values were calculated using the Wilcoxon test. The red dot marks an outlier sample (SBM1148), which was excluded from statistical analyses (see Methods).

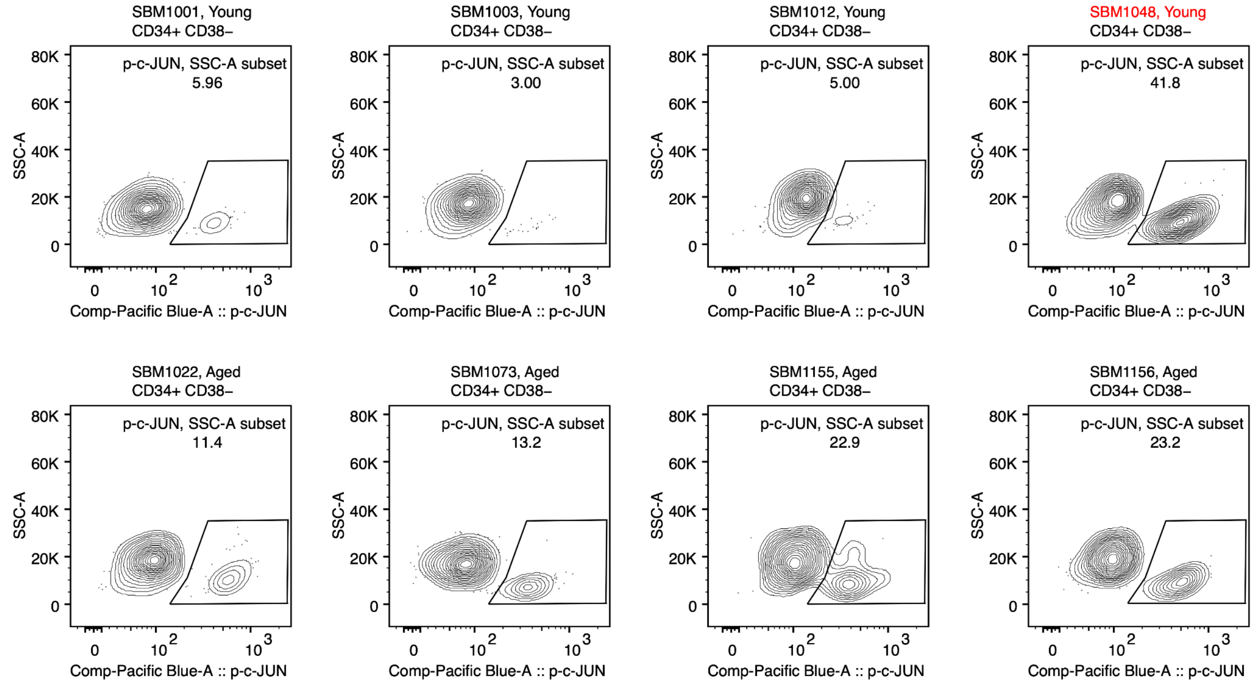

Fig. S12. p-c-JUN population is enriched in aged samples. Gating marks phospho c-JUN-positive population, which tended to be more abundant in aged samples (**Fig. 2i**). The outlier sample is shown in red.

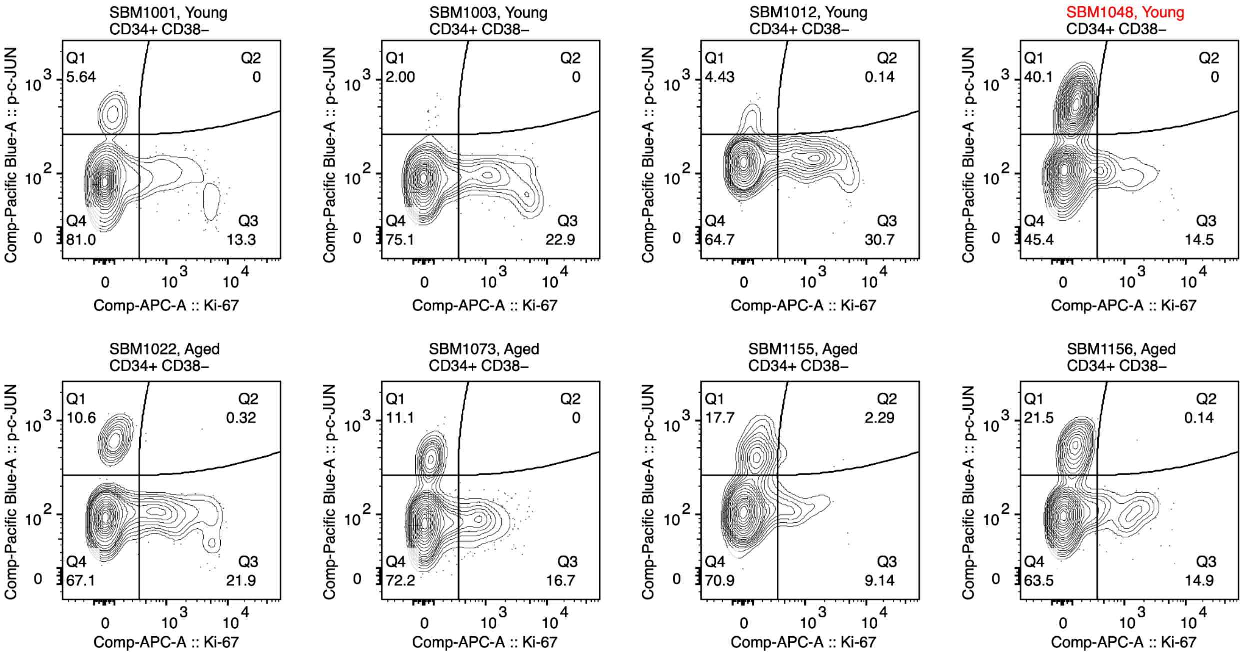

Fig. S13. phospho-c-JUN-positive CD34+CD38- population is mostly quiescent. Gating separates CD34+CD38- population based on p-c-JUN and Ki-67 levels; see **Fig. 2k**. The outlier sample is shown in red.

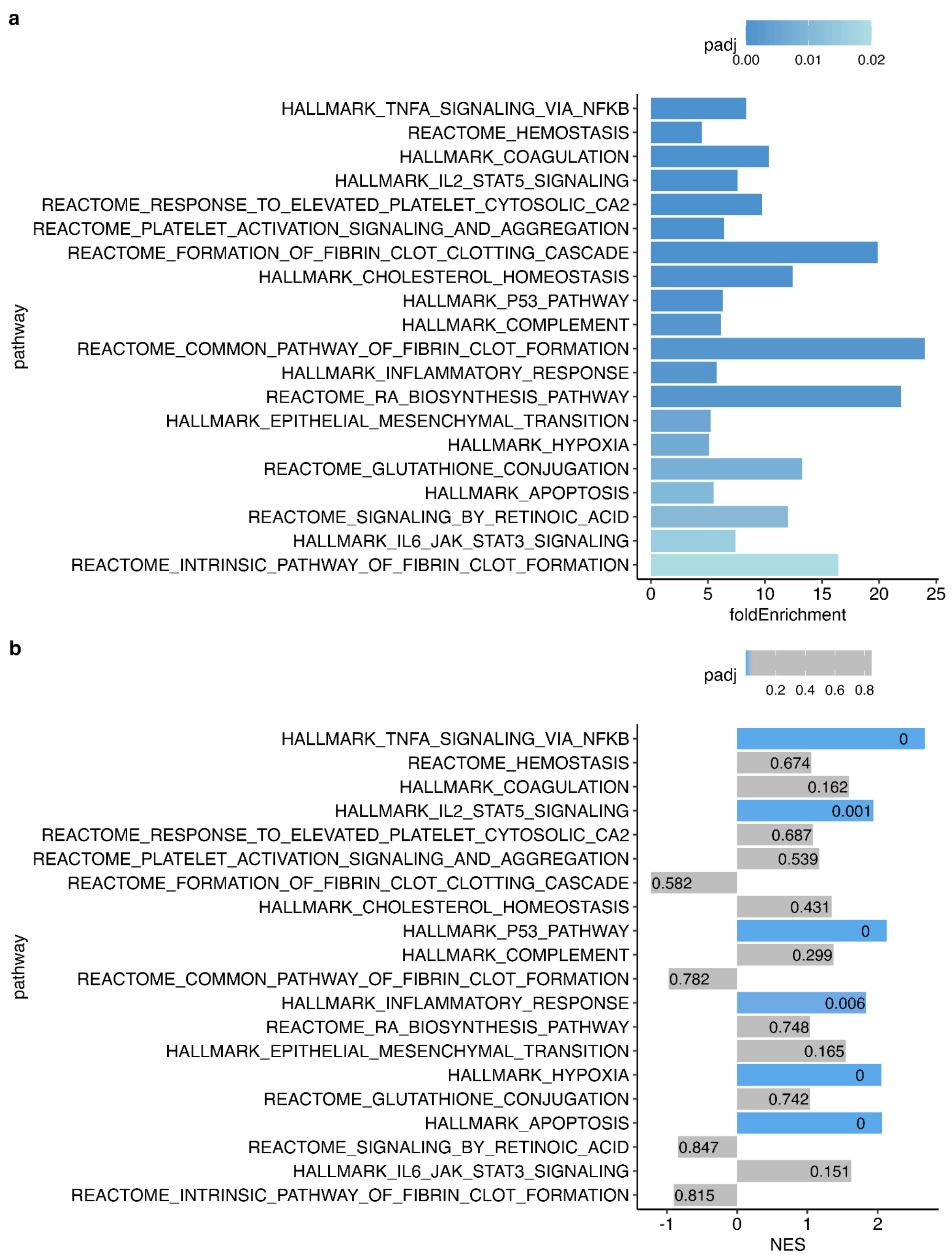

Fig. S14. Over-representation analysis reveals pathways associated with HSC aging in mice. a. Twenty most significantly over-represented pathways are shown, arranged by increasing adjusted p-value. The mouse aging signature from [34] was used. **b.** GSEA results of DE results from this study (human), shown for the same 20 pathways. NES, normalized enrichment score. Numbers show adjusted p-value.

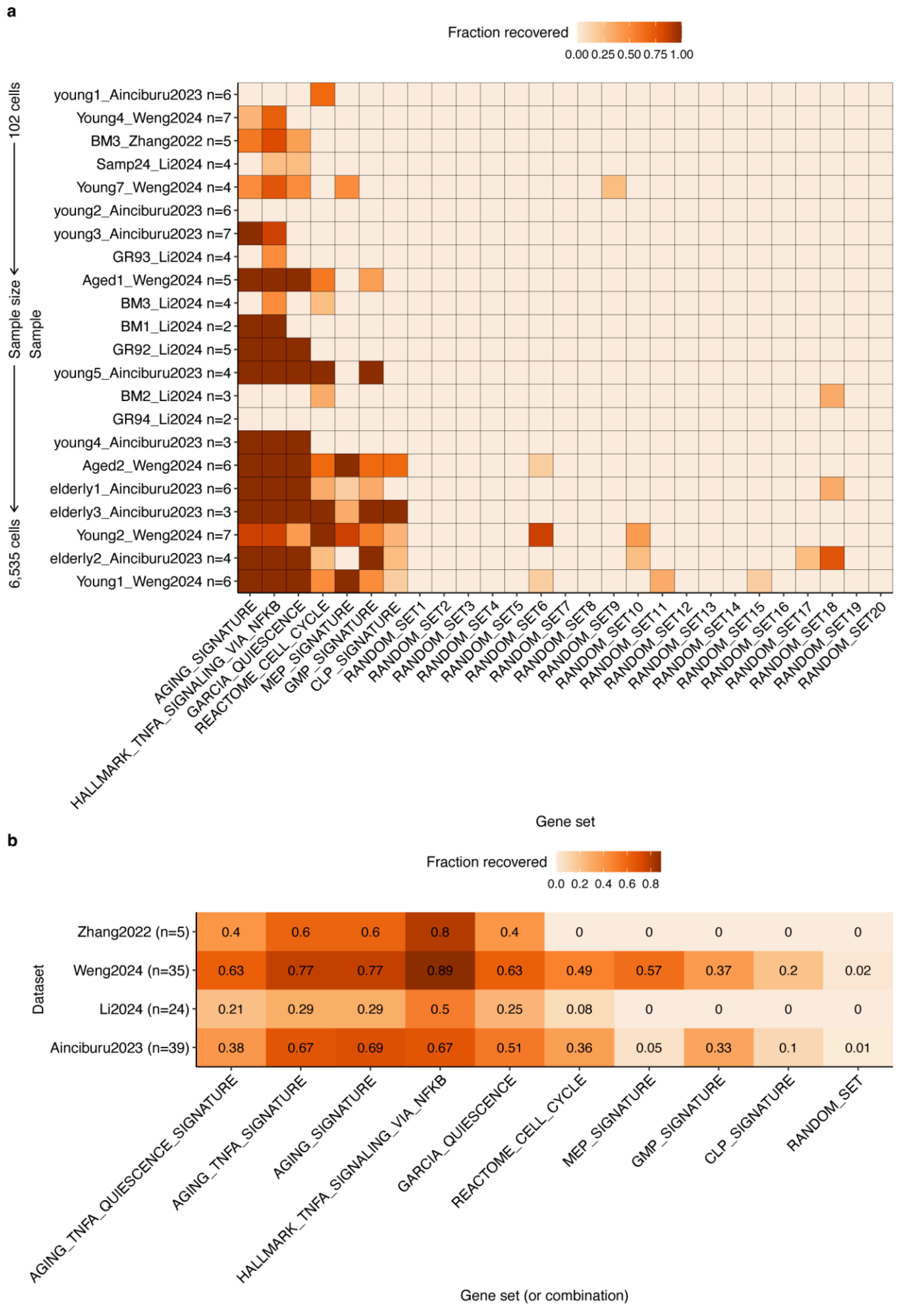

Fig. S15. Functionally relevant gene sets and their combinations are enriched in cNMF programs, compared to 20 random gene sets. a. Fraction of stable cNMF runs that recovered a positively enriched gene set, across 22 samples used for cNMF; samples are ordered by the number of HSCs, ***n*** specifies the number of visually stable cNMF runs per sample (for example, 'Young1_Weng2024 n=6' means that for sample Young1 from dataset Weng2024, there were

six stable cNMF runs among k=3..10 tested). **b.** Recovery rate of gene sets/their combinations, aggregated across datasets; ***n*** specifies the total number of stable cNMF runs per dataset, numbers in tiles show fractions; for the random set, the average fraction across 20 random sets is shown.
