# Supplementary material for: An inflammatory and quiescent HSC subpopulation expands with age in humans
Source: Genome Biol. 2026 Jan 16;27:30. doi: 10.1186/s13059-026-03936-z (PMC12892447; doi:10.1186/s13059-026-03936-z)
Supplement: Supplementary file 3 — Additional file 3. Supplementary note exploring the signal of senescence. [file 13059_2026_3936_MOESM3_ESM.pdf]

Supplementary Note

Is the signal of quiescence related to senescence?

While our data indicate robust age-associated activation of genes related to quiescence, the enrichment of TP53 pathway in aged samples points at possible induction of senescence and permanent cell cycle arrest in aged cells. To address to what extent cell cycle status of aging HSCs has been assessed in the literature, we point to seven studies. (1) Noda *et al.* show slower BrdU uptake by HSCs from old compared to young mice [1]. (2) The Passegué lab showed that aged HSCs re-enter the cell cycle in culture, albeit with slower kinetics than young HSCs, and found low detectability of β-Gal and *CDKN2A* [2]. (3) Kirschner *et al.* identified an age-specific HSC cluster in mice expressing senescence-specific p53 genes and quiescence-supporting factors by single-cell RNA-sequencing of aged mouse HSCs [3]. (4) A meeting abstract by Kovtonyuk *et al.* reports increased quiescence in aged mouse HSCs based on EdU labeling and CFSE dilution [4]. (5) The Geiger lab showed that Ki67 expression does not differ between young and aged human HSCs, but aged HSCs showed delayed cell cycle entry in vitro [5]. (6) Su *et al.* demonstrated that aged mouse HSCs have increased quiescence using Ki-67 staining and decreased proliferation using *in vivo* BrdU labeling [6]. (7) Poisa-Beiro *et al.* analyzed single-cell RNA-sequencing of mouse and human HSPCs, and identified enrichment of senescence signatures in aged cells [7]. However, the rigorous detection of senescent cells is a topic of active discussion in the field [8,9]. Moreover, differentiating between quiescent and senescent states within a single snapshot of time is challenging, if not impossible, as many markers are similarly affected in both senescent and quiescent states [10]. As described below, our observations suggest that senescence is not the only factor contributing to the observed patterns in our data.

Addressing the possible presence of senescence signals in our data, we explored the behaviour of several potentially relevant gene sets in our Aged vs. Young comparison and in the metaGEP1 program that we claimed was associated with quiescence. The selected gene sets included the two quiescence signatures already included in our prior analysis and several senescence-associated gene sets. We also included gene sets representative of p53 signaling, ROS and DNA damage response to assess cellular stress, and the Jak-Stat signaling pathway, which were associated with DNA damage- and proliferation-associated functional declines of mouse HSCs [3,11]. We did not include G0 markers in this analysis as we could not find a gene set signature specific to the G0 state other than the quiescence signatures mentioned above.

To assess senescence signal, we included six human gene sets from MSigDB that may cover different manifestations of the senescence process: "SAUL_SENESCENCE", "FRIDMAN_SENESCENCE", "REACTOME_CELLULAR_SENESCENCE", "REACTOME_OXIDATIVE_STRESS_INDUCED_SENESCENCE", "REACTOME_ONCOGENE_INDUCED_SENESCENCE", and "REACTOME_SENESCENCE_ASSOCIATED_SECRETORY_PHENOTYPE_SASP" (which we term "REACTOME_SASP" in the figures below). We also decided to construct a “consensus” gene set related to senescence, but found no genes shared across all six gene sets. We thus constructed two additional gene sets, named "SHARED_SENESCENCE_GENES_FOUR" and

"SHARED_SENESCENCE_GENES_THREE" that contained genes shared across at least four (16 genes) or three (97 genes) senescence gene sets, respectively.

We started by assessing the enrichment of the gene sets selected for this analysis (n=15) in our pseudobulked differential expression results between young and aged HSCs. **Suppl. Note Fig. 1** shows GSEA results, similarly to **Fig. 1c**. **Suppl. Note Fig. 2** shows the volcano plot presented in **Fig. 1b**, with genes belonging to every selected gene set highlighted.

Except for FRIDMAN_senescence, none of the newly added pathways show significant upregulation in aged samples, including senescence-associated secretory phenotype (SASP) pathways like SAUL_SENESCENCE or REACTOME_SASP (**Suppl. Note Fig. 1**). There is also no signal of enrichment for DNA damage response or ROS pathway gene sets. The gene set members that are most significantly upregulated in Aged samples are often AP-1 genes, while most of the remaining gene set members remain largely unchanged in expression (**Suppl. Note Fig. 2**). Of the 97 genes present in 3/6 senescence-related gene sets, only three genes are upregulated, including two AP-1 genes. This suggests that in our data, there is no robust signal of senescence that could explain the observed quiescence signature enrichment.

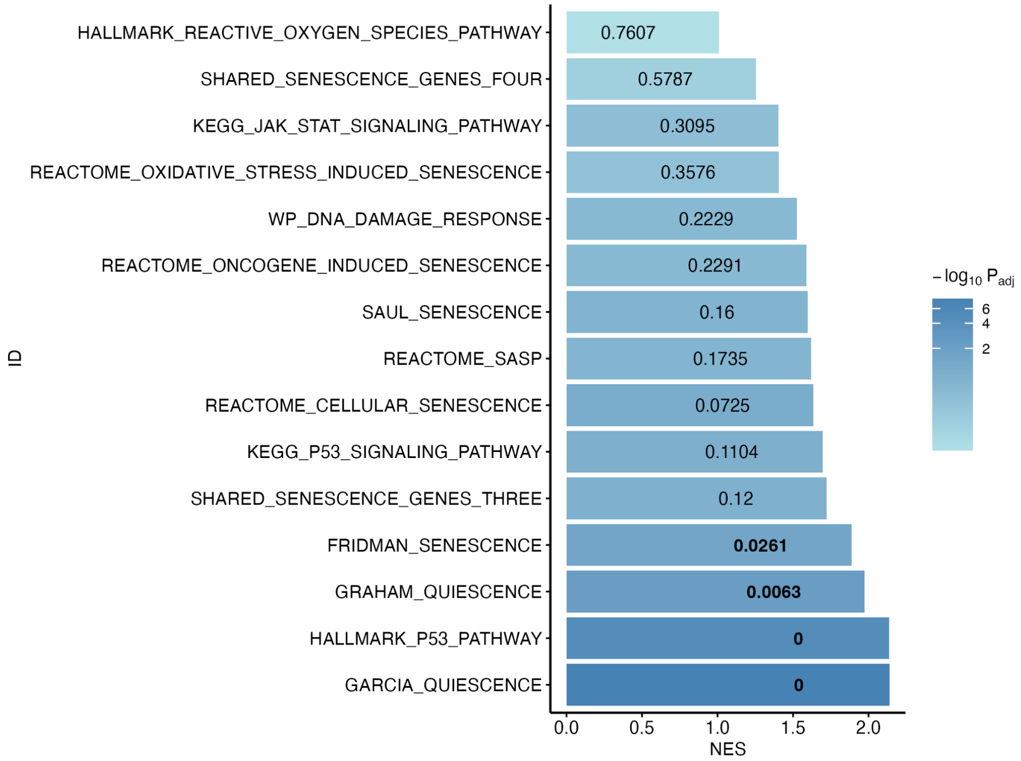

Supplementary Note Figure 1. Enrichment of the selected gene sets in differential expression results of aged vs young HSCs. Numbers in bars show adjusted p-values, with values below 0.05 shown in bold.

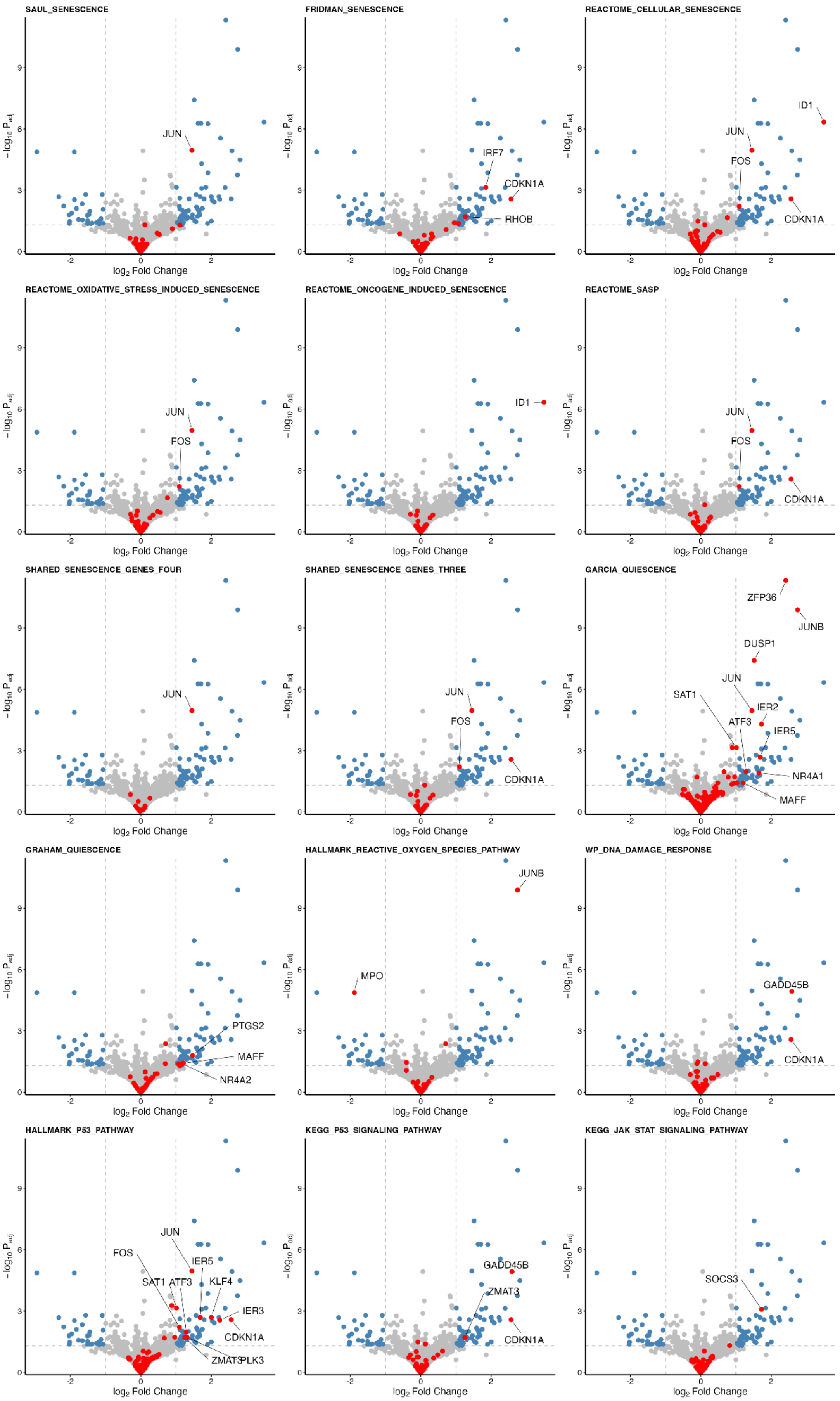
SAUL_SENESCENCE
JUN
FRIDMAN_SENESCENCE
IRF7
CDKN1A
RHOB
REACTOME_CELLULAR_SENESCENCE
ID1
JUN
FOS
CDKN1A
REACTOME_OXIDATIVE_STRESS_INDUCED_SENESCENCE
JUN
FOS
REACTOME_ONCOGENE_INDUCED_SENESCENCE
ID1
REACTOME_SASP
JUN
FOS
CDKN1A
SHARED_SENESCENCE_GENES_FOUR
JUN
SHARED_SENESCENCE_GENES_THREE
JUN
FOS
CDKN1A
GARCIA_QUIESCENCE
ZFP36
JUNB
DUSP1
JUN
SAT1
IER2
ATF3
IER5
NR4A1
MAFF
GRAHAM_QUIESCENCE
PTGS2
MAFF
NR4A2
HALLMARK_REACTIVE_OXYGEN_SPECIES_PATHWAY
JUNB
MPO
WP_DNA_DAMAGE_RESPONSE
GADD45B
CDKN1A
HALLMARK_P53_PATHWAY
JUN
FOS
IER5
SAT1
ATF3
KLF4
IER3
CDKN1A
ZMAT3
PLK3
KEGG_P53_SIGNALING_PATHWAY
GADD45B
ZMAT3
CDKN1A
KEGG_JAK_STAT_SIGNALING_PATHWAY
SOCS3
$-\log_{10} P_{adj}$
$\log_2$ Fold Change

Supplementary Note Figure 2. Volcano plot shows the differentially expressed genes in Young vs Aged samples (identical to Fig. 1b). Each panel highlights members of a certain gene set (labeled above) in red, with significant hits labeled (fold change > 1, P<0.05).

While the key cell cycle arrest player p53 is not significantly affected by age, p21 (*CDKN1A*), often used as a marker of a cell cycle arrest, is upregulated in aged samples and is present in several selected pathways, including three senescence-associated gene sets. p21 upregulation is mostly driven by the Zhang2022 dataset and has low expression levels in the remaining datasets (**Suppl. Note Fig. 3**), which may indicate that aged Zhang samples contain more senescent cells. At the same time, p21 accumulates in both senescent cells and quiescent cells between cell cycles [10]. Additionally, it was implicated in quiescence in HSCs [12] and fibroblasts [13]. We did not detect expression of the more canonical senescence marker *CDKN2A*, consistent with earlier studies showing its low expression in bone marrow [7,14]. Altogether, the observed p21 upregulation does not seem to robustly map senescence in aged samples.

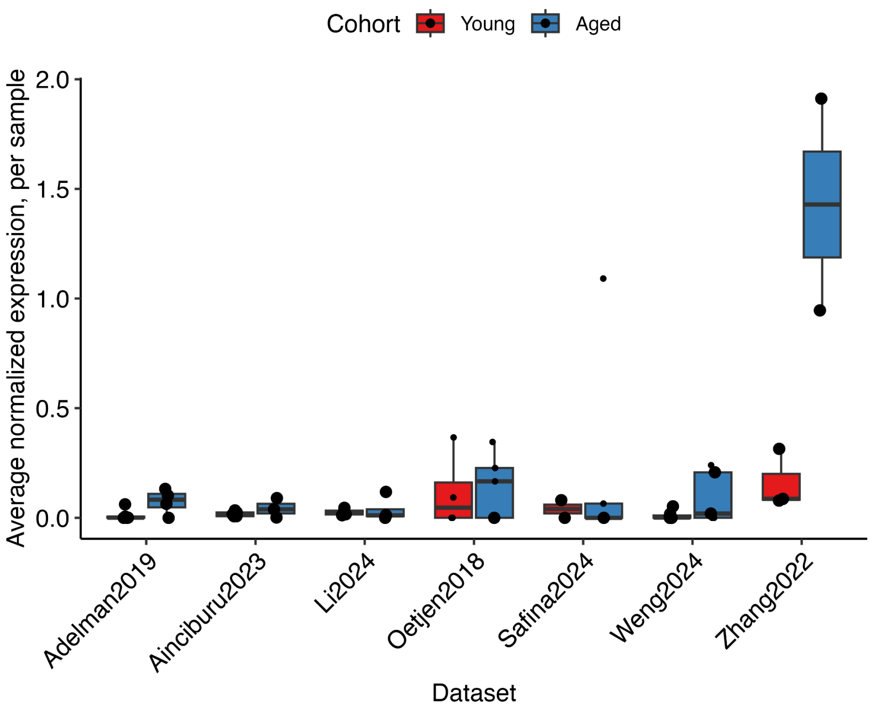

Supplementary Note Figure 3. Average normalized expression of CDKN1A (p21) in young and aged samples. Larger points show samples with at least 20 cells.

A prior report described a sub-population of aged HSCs in mice that showed enrichment of senescence-specific p53 genes and Jak/Stat-signaling [3], which could relate to a DNA damage checkpoint and selection of myeloid-biased HSCs [11]. However, we did not observe prominent upregulation of DNA damage response or Jak/Stat-signaling (**Suppl. Note Fig. 2**). This may suggest that healthy aged human HSCs do not undergo DNA damage and senescence to the

same extent as aged mouse or MDS samples [11], so that our current dataset is not powered to capture this axis.

Finally, since the enrichment of the quiescence signature is specific to metaGEP1, we also checked for the enrichment of the selected pathways in metaGEPs 1-4 (**Suppl. Note Fig. 4**). Except for the pathways already present in **Fig. 2** of the main text, none of the gene sets showed significant enrichment in the metaGEP1 program, again suggesting that the quiescence signal is unlikely to be explained by senescence alone.

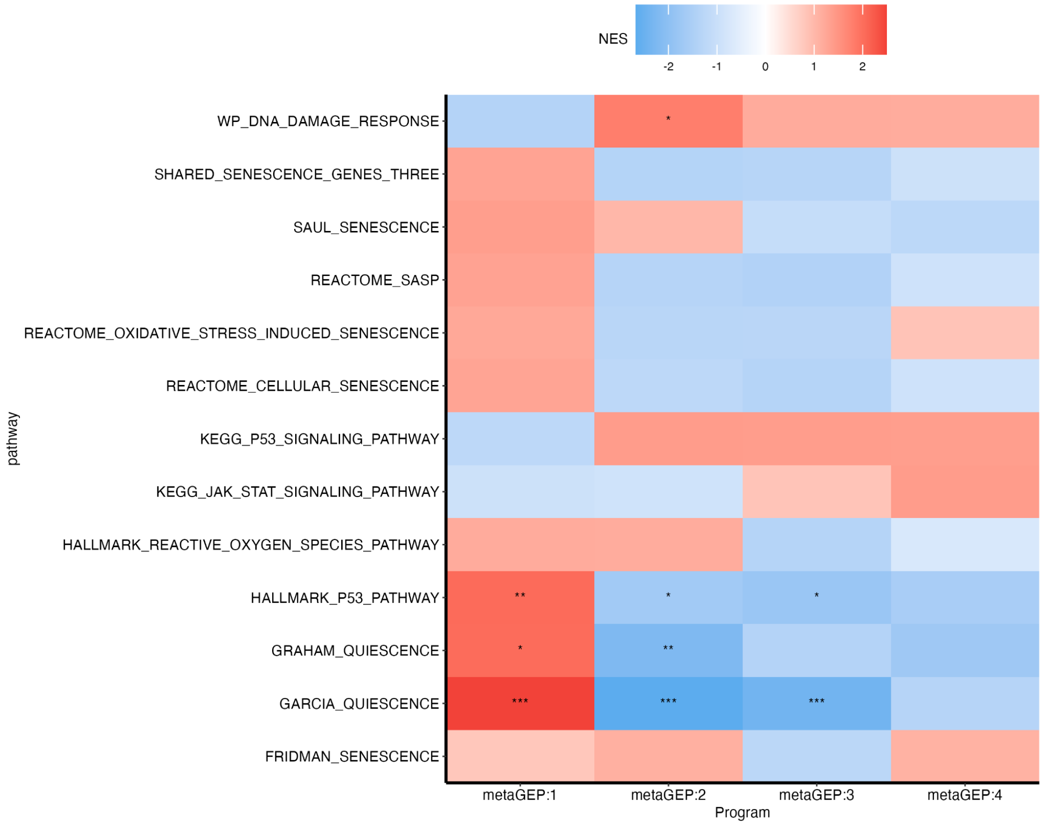

Supplementary Note Figure 4. Heatmap shows the enrichment of the selected gene sets in metaGEPs as inferred by GSEA. BH-corrected p-values: *, p<0.05, **, p<0.01, ***, p<0.001.

To summarize, while we cannot attribute all of the observed signal to quiescence only, we conclude that quiescence (temporal cell cycle arrest) does contribute to our observations in Aged vs Young comparisons and in metaGEP1 program.

References

1. Noda S, Ichikawa H, Miyoshi H. Hematopoietic stem cell aging is associated with functional decline and delayed cell cycle progression. Biochem Biophys Res Commun. 2009;383:210–5.

2. Flach J, Bakker ST, Mohrin M, Conroy PC, Pietras EM, Reynaud D, et al. Replication stress is a potent driver of functional decline in ageing haematopoietic stem cells. Nature. 2014;512:198–202.

3. Kirschner K, Chandra T, Kiselev V, Flores-Santa Cruz D, Macaulay IC, Park HJ, et al. Proliferation Drives Aging-Related Functional Decline in a Subpopulation of the Hematopoietic Stem Cell Compartment. Cell Rep. 2017;19:1503–11.

4. Kovtonyuk LV, Ashcroft P, Spaltro G, Tata NR, Skoda RC, Bonhoeffer S, et al. Hematopoietic stem cells increase quiescence during aging. Blood. 2019;134:2484–2484.

5. Amoah A, Keller A, Emini R, Hoenicka M, Liebold A, Vollmer A, et al. Aging of human hematopoietic stem cells is linked to changes in Cdc42 activity. Haematologica. 2022;107:393–402.

6. Su T-Y, Hauenstein J, Somuncular E, Dumral Ö, Leonard E, Gustafsson C, et al. Aging is associated with functional and molecular changes in distinct hematopoietic stem cell subsets. Nat Commun. 2024;15:7966.

7. Poisa-Beiro L, Landry JJM, Yan B, Kardorff M, Eckstein V, Villacorta L, et al. A Senescent Cluster in Aged Human Hematopoietic Stem Cell Compartment as Target for Senotherapy. Int J Mol Sci. 2025;26. Available from: http://dx.doi.org/10.3390/ijms26020787

8. Suryadevara V, Hudgins AD, Rajesh A, Pappalardo A, Karpova A, Dey AK, et al. SenNet recommendations for detecting senescent cells in different tissues. Nat Rev Mol Cell Biol. 2024;25:1001–23.

9. Reimann M, Lee S, Schmitt CA. Cellular senescence: Neither irreversible nor reversible. J Exp Med. 2024;221. Available from: http://dx.doi.org/10.1084/jem.20232136

10. Ashraf HM, Fernandez B, Spencer SL. The intensities of canonical senescence biomarkers integrate the duration of cell-cycle withdrawal. Nat Commun. 2023;14:4527.

11. Wang J, Sun Q, Morita Y, Jiang H, Groß A, Lechel A, et al. A Differentiation Checkpoint Limits Hematopoietic Stem Cell Self-Renewal in Response to DNA Damage. Cell. 2014;158:1444.

12. Cheng T, Rodrigues N, Shen H, Yang Y, Dombkowski D, Sykes M, et al. Hematopoietic stem cell quiescence maintained by p21cip1/waf1. Science. 2000;287:1804–8.

13. Perucca P, Cazzalini O, Madine M, Savio M, Laskey RA, Vannini V, et al. Loss of p21 CDKN1A impairs entry to quiescence and activates a DNA damage response in normal fibroblasts induced to quiescence. Cell Cycle. 2009;8:105–14.

14. Saul D, Kosinsky RL, Atkinson EJ, Doolittle ML, Zhang X, LeBrasseur NK, et al. A new gene set identifies senescent cells and predicts senescence-associated pathways across tissues. Nat Commun. 2022;13:4827.
